# Bio-chelate assisted leaching for enhanced heavy metal remediation in municipal solid waste compost

**DOI:** 10.1038/s41598-024-65280-1

**Published:** 2024-06-20

**Authors:** Srishti Khare, Anupam Singhal, Srinivas Rallapalli, Anant Mishra

**Affiliations:** 1 Department of Civil Engineering, Birla Institute of Technology and Science, Pilani, Rajasthan India; 2 Department of Bioproducts and Biosystems Engineering, University of Minnesota, Twin Cities, USA

**Keywords:** Biodegradable chelator, Compost, Circular economy, Heavy metal removal, Optimization, Civil engineering, Environmental impact, Environmental monitoring, Pollution remediation

## Abstract

Municipal solid waste compost, the circular economy's closed-loop product often contains excessive amounts of toxic heavy metals, leading to market rejection and disposal as waste material. To address this issue, the study develops a novel approach based on: (i) utilizing plant-based biodegradable chelating agent, l-glutamic acid, *N*,*N*-diacetic acid (GLDA) to remediate heavy metals from contaminated MSW compost, (ii) comparative assessment of GLDA removal efficiency at optimal conditions with conventional nonbiodegradable chelator EDTA, and (iii) enhanced pre- and post-leaching to evaluate the mobility, toxicity, and bioavailability of heavy metals. The impact of treatment variables, such as GLDA concentration, pH, and retention time, on the removal of heavy metals was investigated. The process was optimized using response surface methodology to achieve the highest removal effectiveness. The findings indicated that under optimal conditions (GLDA concentration of 150 mM, pH of 2.9, retention time for 120 min), the maximum removal efficiencies were as follows: Cd-90.32%, Cu-81.96%, Pb-91.62%, and Zn-80.34%. This process followed a pseudo-second-order kinetic equation. Following GLDA-assisted leaching, the geochemical fractions were studied and the distribution highlighted Cd, Cu, and Pb's potential remobilization in exchangeable fractions, while Zn displayed integration with the compost matrix. GLDA-assisted leaching and subsequent fractions illustrated transformation and stability. Therefore, this process could be a sustainable alternative for industrial applications (agricultural fertilizers and bioenergy) and social benefits (waste reduction, urban landscaping, and carbon sequestration) as it has controlled environmental footprints. Hence, the proposed remediation strategy, chemically assisted leaching, could be a practical option for extracting heavy metals from MSW compost, thereby boosting circular economy.

## Introduction

Municipal solid waste (MSW) generation has increased to nearly 2 billion tonnes annually, increasing contamination risk due to unavailability of sustainable treatment methods^1,2^. MSW management is increasingly moving towards a circular economy, aiming to reduce waste and maximize its use while minimizing environmental impact^3^. In this system, stakeholders place high value on green products, and companies strive to balance profitability with social welfare and environmental considerations^4^. Thus, MSW compost would have great acceptance^5^. European Commission introduced circularity wherein the by-products from one manufacturing process could be employed as a source of secondary raw materials in another^6^. As a result, composting has the benefit of transforming MSW materials into humus-like compounds using bacterial processes. This process can assist municipalities in sustainable waste disposal and creation of valuable products^7^. The resulting compost is a form of organic fertilizer that reduces the demand for pesticides and synthetic fertilizers, improves soil physical and biological properties, and detoxifies polluted soil^8,9^.

Despite its advantages, presence of toxic heavy metals in MSW compost as a result of household hazardous waste being deposited in municipal landfills limits its value^10,11^. This can be confirmed by referring to the microstructural characterization section in our previous study^12^. Heavy metals are extremely toxic even in small amounts, remaining persistent and posing a major risk to both the environment and human health in agricultural soil environments^13,14^. The presence of contaminated MSW compost can lead to a decline in soil fertility and the quality of crops, resulting in substantial adverse effects on soil ecosystems, food security, human health, and economic stability^15,16^.

While the overall contamination levels of heavy metals in MSW compost provide valuable insights^17,18^, prior studies have shown that the metal stability, leachability, and ecological toxicity of trace metals depend significantly on their geochemical fractions^19,20^. Heavy metals exhibit unique environmental characteristics depending on their states, such as solubility in water, exchangeability, association with carbonates, occlusion in Mn–Fe oxides, binding to organic matter, or occurrence in residual forms. It is vital to comprehend these differences through sequential extractions for accelerating the contaminated MSW compost^21^. Most studies on MSW compost have primarily concentrated on the overall concentrations of heavy metals as treating the contaminated MSW compost presents significant challenges^12^. There is a growing need for innovative and economical approaches to facilitate the faster and more effective decontamination of contaminated MSW compost.

Previous studies have specifically focused on reducing the impact of heavy metals in soil and sewage sludge through methods such as electrokinetic remediation^22,23^, phytoremediation^24^, chemical-assisted leaching^25^, bioremediation^26^, and immobilization^27^. The comparison of these remediation technologies, operational mechanisms, advantages, and limitations are mentioned in Table 1. Among these approaches, chemical-assisted leaching has gained prominence for its straightforward operation, quick results, and ability to permanently remove heavy metals from contaminated soils, sludge, and compost in recent years^12,16,23^. The efficiency of leaching in eliminating metal pollutants relies on the selection of a suitable chemical agent or extractant. Hence, choosing the appropriate chemical agents is essential for successful removal of heavy metals^28,29^. Chelating agents are commonly used as extractants in soil washing due to their strong capacity to create highly soluble and stable complexes with metal ions among the leaching agents^30,31^. The aminopolycarboxylate chelator ethylenediaminetetraacetic acid (EDTA) is widely used for its strong ability to bind various heavy metals. In our previous study^12^, we demonstrated EDTA's excellent efficiency in extracting Cd, Cu Pb, Ni, and Zn from contaminated MSW compost. However, EDTA's poor biodegradability and long-lasting presence in the environment could lead to secondary contamination and ecological worries^32^.

**Table 1:** Operational mechanisms, advantages, and limitations of heavy metal removal techniques.

Remediation technology	Operational mechanism	Advantages	Limitations	References
Physical remediation				
Surface capping	Physical containment	Easy to install, low in cost, high security	Limited to small areas and certain geographic locations, loss of land cropping function	^33^
Encapsulation	Physical containment and isolation	High security, fast to install	Limited to small, shallow contamination areas, high cost, loss of land cropping function	^33, 34^
Chemical remediation				
Chemical leaching	Contaminant removal by chemical solutions, and mechanical separation	High efficiency, fast effects, low cost, simple to install; suitable for severely contaminated matrix; minimal physiochemical disturbance	Best for coarse-textured matrix with high permeability; use of leaching agents incorrectly can result in pollution	^33, 35, 36^
Chemical stabilization	Contaminant deactivation by physiochemical transformation	Low-cost, effective, and convenient	Metal-specific, pollutants cannot be removed thoroughly, temporary effectiveness	^37, 38^
Electrokinetic remediation	Contaminant removal by electricity	Contaminant removal, minimal physiochemical disturbance	Time-consuming, low efficiency, best for a fine-textured matrix with low permeability, the physiochemical properties are changed	^29, 39^
Biological remediation	Contaminant transformation by microorganisms	Low cost, simple to implement, minimal physiochemical disturbance	Low efficiency, high specificity, a certain concentration limit, difficult to implement at site	^40, 41^
Phytoremediation	Contaminant removal and/or stabilization by plants	High public acceptance, low cost, easy to implement, suitable for large and low contamination areas	Limited to shallow contamination, metal-specific, time-consuming, low efficiency	^42, 43^
Photocatalytic degradation/ treatment	Contaminant removal using photocatalyst	It is possible for several forms of contamination, particularly in mixed contaminated media	Time-consuming, highly reliant on power supply, incorrect supply of nutrients might have adverse effects on the ecosystem	^41, 44^

Consequently, practitioners are focusing on biodegradable chelators as sustainable and environmentally friendly options with reduced potential for secondary contamination^32,45^. l-glutamate-*N*,*N*′-diacetic acid, a biodegradable chelator derived from plant sources, exhibits remarkable chelating abilities and superior biodegradability, with over 80% decomposing within 28 days^46–48^. Additionally, it poses fewer ecological risks when compared to traditional chelators. In addition, the special composition of GLDA enables it to selectively attach to heavy metal ions, reducing the chance of nutrient leaching and thus maintaining soil fertility, while also promoting greater plant growth^48^. In recent times, several studies have been released discussing soil washing techniques for the treatment of hazardous contaminants^47,49^. These studies have presented evidence of the significant efficacy of both pure complexing agent GLDA and its combinations with citric acid, ascorbic acid, or Nitrilotriacetic acid (NTA), [S,S]-stereoisomer of ethylenediaminedisuccinic acid EDDS, among others, in eliminating heavy metals while preserving most nutrients in the soil. Recycling GLDA for this purpose was also a significant area of study, and its efficiency was discovered to be similar to that of other solutions^50^. The increasing use of GLDA in various applications has prompted further research into decontaminating MSW compost^51^.

**Figure 1:**
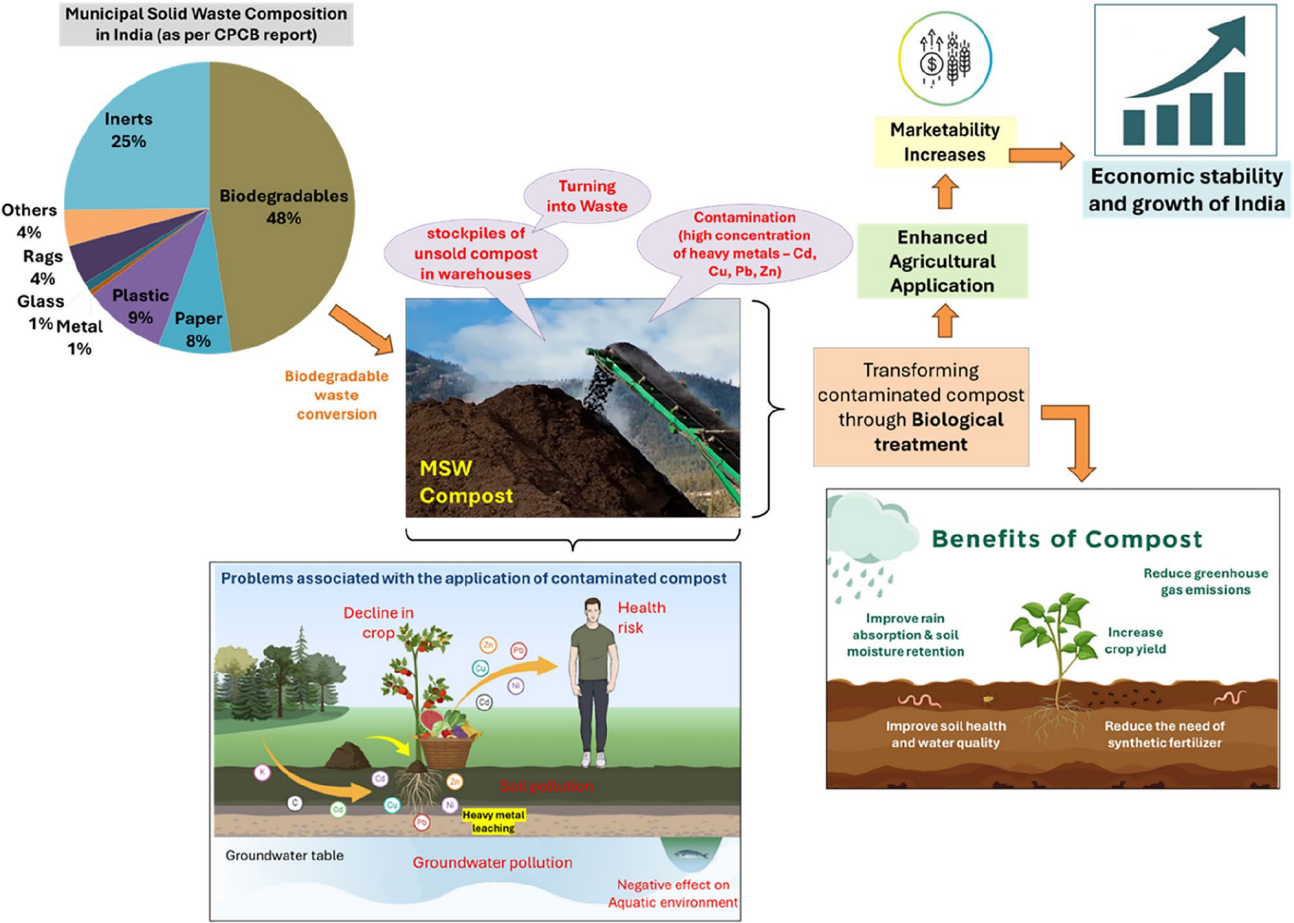
Overview of the challenges and potential solutions for transforming contaminated municipal solid waste compost into a marketable product.

Figure 1 provides an overview of the challenges and potential solutions related to transforming contaminated MSW compost. There is a significant gap in the research literature on using l-glutamate-*N*,*N*′-diacetic acid (GLDA) to treat heavy metal-contaminated municipal solid waste (MSW) compost, a challenge that affects its quality and marketability. The research hypothesis proposes that GLDA-assisted leaching can effectively reduce the high heavy metal content in MSW compost in a biodegradable manner. This treatment could enhance the compost's quality, marketability and reduce ecological impact, thereby preventing it from remaining unsold and accumulating as waste in warehouses, a situation that currently discourages farmers from using it. Consequently, this approach would foster economic stability and environmental sustainability. The novel aspects of this study are as follows:

1. Use of plant-based l-glutamate-*N*,*N*′-diacetic acid (GLDA) as an environmentally friendly (biodegradable) chelant for extracting heavy metals from contaminated MSW compost to boost circular economy.
2. Comparative assessment of efficiencies of GLDA and conventionally used chelator EDTA to remediate heavy metal contamination from MSW compost.
3. Advancing sustainable remediation by optimizing GLDA-assisted leaching and enhancing the understanding of heavy metal mobility and bioavailability patterns in MSW compost

This study focuses on (i) assessing the effectiveness of the biodegradable chelator GLDA as an environmentally friendly alternative to EDTA for extracting heavy metals using a sequential batch process; (ii) exploring how process parameters impact removal efficiency and designing optimal treatment conditions to improve sustainability in remediation efforts; and (iii) examining geochemical fractionation to gain insights on mobility and bioavailability of associated heavy metals. Additionally, the research complies with applicable laws and proposes a feasible, cost-effective, and eco-friendly approach for MSW compost remediation to enhance its role in the circular economy.

## Material and methodologies

**Figure 2:**
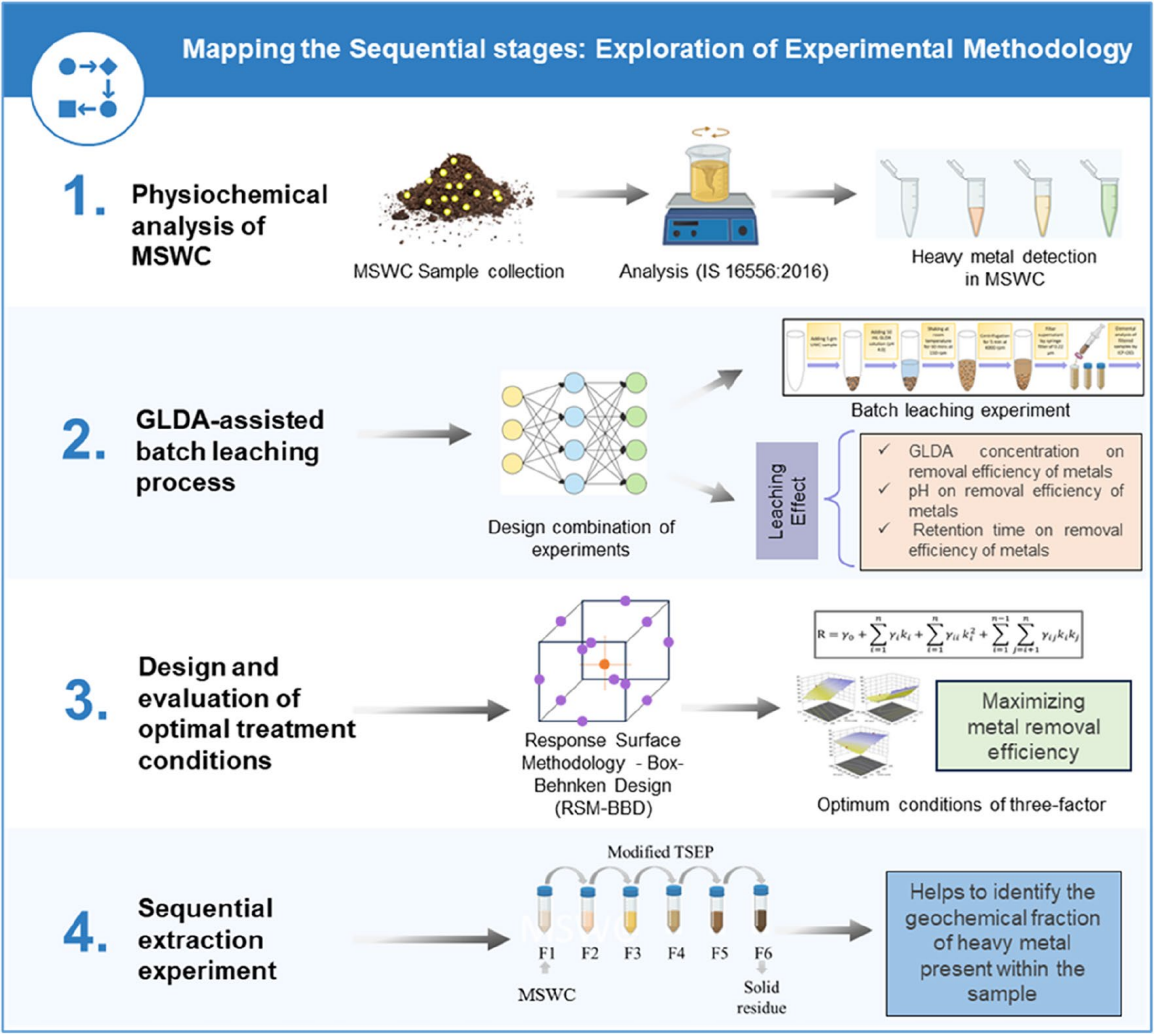
Methodology chart depicting the research approach used to conduct the study.

This study concentrates on decontaminating polluted MSW compost with the use of biodegradable chelating agents GLDA. Figure 2 depicts the flowchart of the research process, demonstrating the importance of each step involved in the investigation. Datasets used/analysed in the study are provided in Table 2 and Supplementary material (Tables S1–S3).

**Table 2:** Change in physicochemical properties and metal content of the examined MSW compost samples in both pre- and post-leaching treatment (Optimum condition: 150 mM, pH 2.9, and retention time 120 min).

Property	Unit	Requirement	MSW compost		
			Original	GLDA treated	EDTA treated
pH		6.5–7.5	6.4 ± 0.04	6.32 ± 0.04	6.18 ± 0.22
Conductivity	ds m^−1^	≤ 4	2.2 ± 0.2	1.79 ± 0.08	1.63 ± 0.21
Bulk density	g cm^−3^	0.7–0.9	0.64 ± 0.58	0.61 ± 0.09	0.61 ± 0.15
Total organic carbon	% by mass	≥ 14	27.13 ± 0.85	22.34 ± 1.59	22.45 ± 1.77
Total nitrogen	% by mass	≥ 0.8	1.71 ± 0.05	1.22 ± 0.01	1.14 ± 0.02
Total phosphorus	% by mass	≥ 0.4	0.41 ± 0.02	0.17 ± 0.02	0.21 ± 0.03
Total potassium	% by mass	≥ 0.4	0.18 ± 0.01	0.05 ± 0.01	0.19 ± 0.02
Cadmium (as Cd)	mg kg^−1^	≤ 5	21.80 ± 1.46	2.11 ± 0.05	1.29 ± 0.02
Copper (as Cu)	mg kg^−1^	≤ 300	1836.51 ± 11.65	331.30 ± 8.16	323.96 ± 11.41
Lead (as Pb)	mg kg^−1^	≤ 100	698.35 ± 8.65	58.52 ± 4.3	47.76 ± 2.33
Zinc (as Zn)	mg kg^−1^	≤ 1000	8130.73 ± 42.21	1598.50 ± 20.45	1159.44 ± 8.82

Experimental results are reported as mean ± standard deviation (n = 3).

### MSW compost sampling and physicochemical analysis

MSW compost was obtained from a Delhi Municipal Corporation compost plant in India. The sample was collected according to the guidelines specified in the IS 16,556:2016^52^ standard, which governs the production of manure-grade compost from municipal solid waste. The physicochemical parameters such as pH, electrical conductivity, bulk density, total organic content, total nitrogen, phosphorus, and potassium were performed on collected sample. Metal concentration analysis in the samples was conducted using the AVIO200 ICP-OES technique, which involves Inductively Coupled Plasma and Optical Emission Spectroscopy. The detailed assessment of physicochemical properties for the collected MSW compost is presented in Supplementary Material S1. MSW compost exhibits high water content. Therefore, in the treatment process, MSW compost was placed in an oven at 70 °C for 24 h. According to the standard IS 16,556:2016 (Municipal Solid Waste Compost, Manure Grade- Specification), this procedure for analysing moisture content ensures that the sample reaches equilibrium by keeping the compost sample at 70 °C for 24 h and all moisture content is removed. Thus, there is no remaining moisture content in the MSW compost before treatment with GLDA. Subsequently, varying dosages of GLDA were used to ensure effective treatment and higher efficiency.

### Biodegradable chelator: GLDA

**Figure 3:**
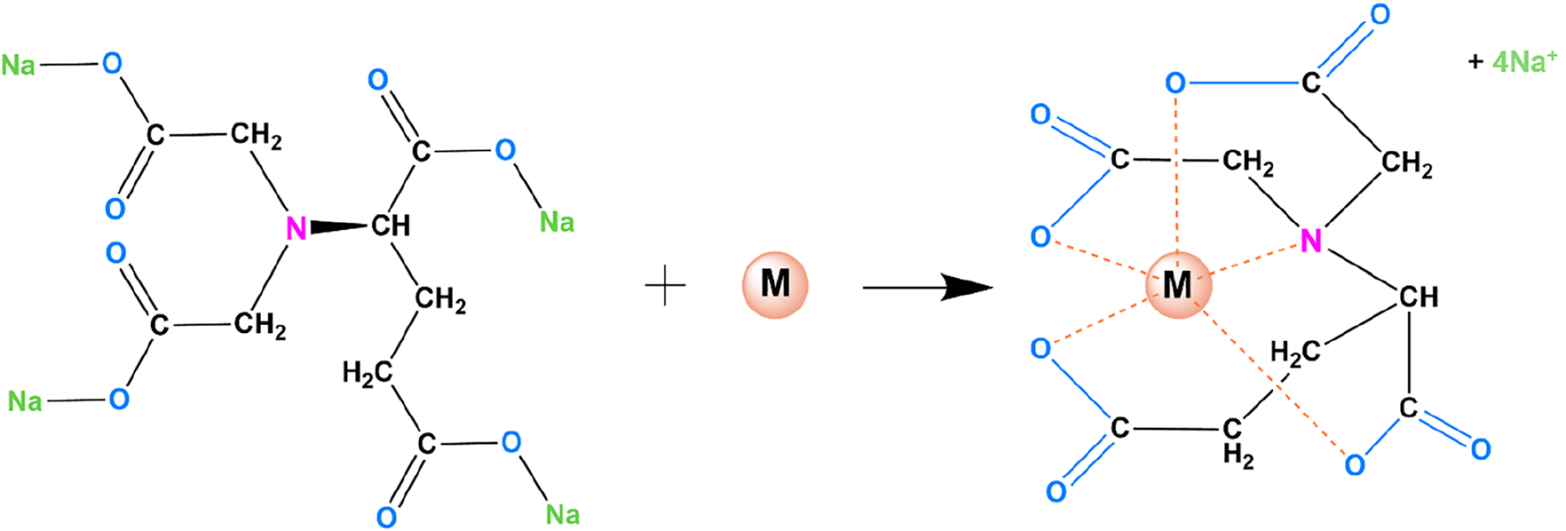
Symbolic diagram of bonding between a metal ion and GLDA complex.

The study utilized the biodegradable chelating agent GLDA, which was obtained from Aquapharm, India. It has a molecular weight of 351.1 g/mol, density of 1320 kg/m^3^, solid content of 38%, and a purity equal to or greater than 99.9%. As for the comparison with non-biodegradable synthetic chelator EDTA purchased from CDH Pvt. Ltd., it has a molecular weight of 372.24 g/mol and a purity level equal to or higher than 99.50%. Figure 3 depicts the bonding between a heavy metal ion and GLDA complex.

### GLDA-assisted leaching treatment

**Figure 4:**
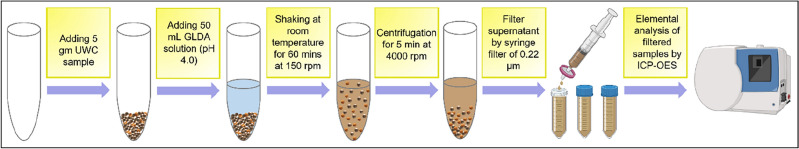
Schematic representation of GLDA-assisted batch leaching process.

An experimental setup was developed using polyethylene tubes cleaned with acid, following a one-variable-at-a-time approach. This arrangement aimed to examine the effects of GLDA concentration, pH, and retention time on the removal of Cd, Cu, Pb, and Zn from contaminated MSW compost. Each run involved mixing a 5 g MSW compost sample with 50 mL GLDA solution in acid-cleaned polyethylene tubes (~ 100 mL). The mixture was then agitated at 150 rpm in an orbital shaker under ambient temperature conditions. Varying concentrations of GLDA (ranging from 1.00 to 200.00 mM) were tested for their impact on metal removal while maintaining consistent conditions: pH at 4.0 and a retention time of 60 min. Additionally, different pH levels (ranging from 2.0 to10.) were examined to assess their effect on metal removal efficiency by adjusting the pH through the addition of HNO_3_ or/and NaOH solution while keeping other variables constant (160 mM GLDA concentration and a retention time of 60 min). Figure 4 depicts a simplified visual representation of an in-house batch leaching process. In addition, the kinetic investigation included exposing the samples to various durations (ranging from 5 to 480 min) under constant experimental conditions, with GLDA concentration and pH set at 160 mM and 3.0 respectively. Moreover, Milli-Q water was consistently used throughout the experiment for precise control. Following centrifugation (5 min, 4000 rpm) and filtration through a 0.22 μm membrane filter, the leachate sample was analyzed for metal concentrations using ICP-OES. Prior to analysis, the solutions were appropriately stored at 4 °C. To assess the kinetics involved in metal removal, integration of the second-order kinetic model outlined in S2 was carried out.

### Modelling and optimization of heavy metal leaching conditions using Response surface method

**Figure 5:**
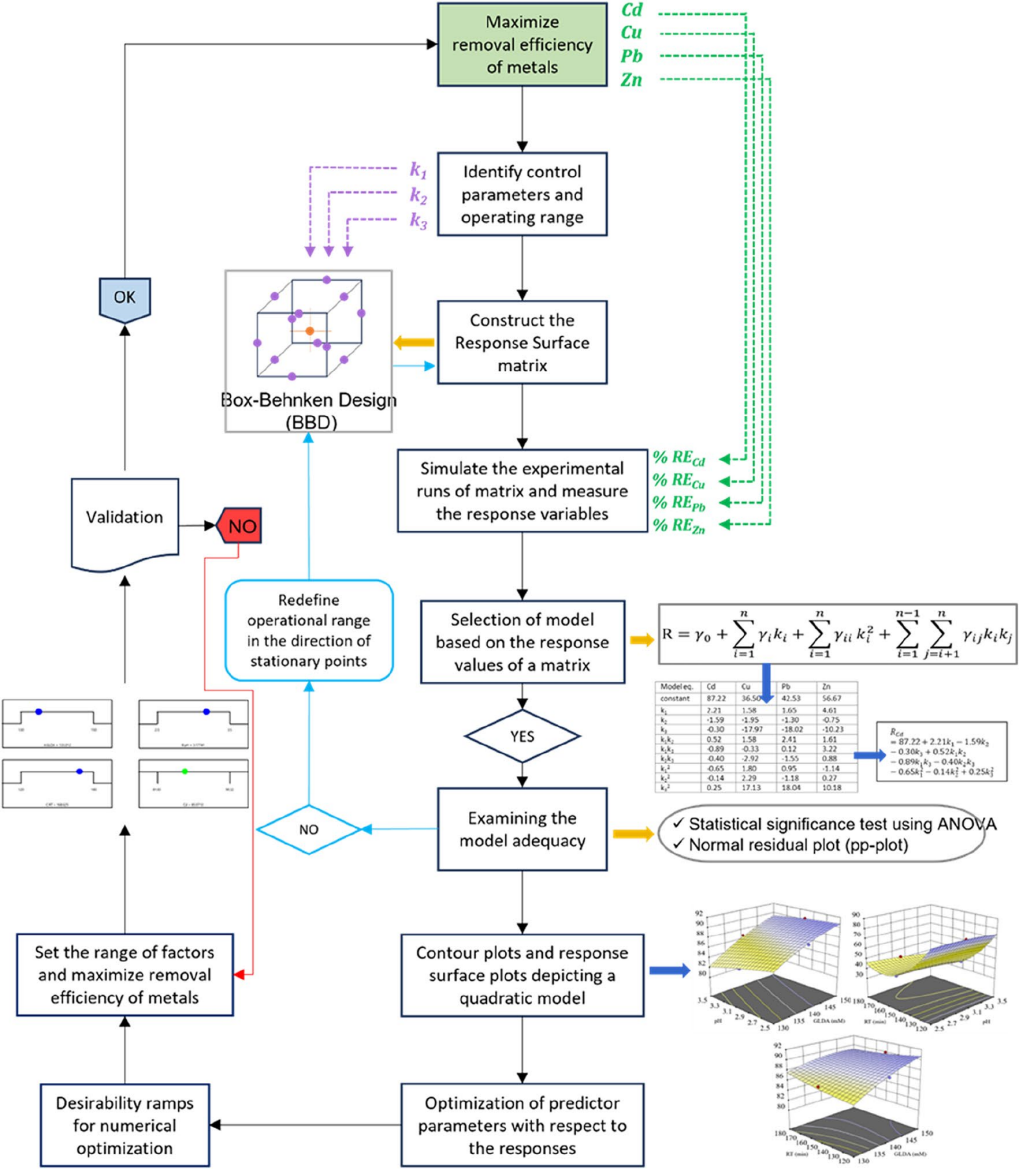
Concept and result interpretation of RSM optimization.

Response surface method (RSM) had been used to design and optimize the operational parameters for the system that meets the operational specifications by using the software Design-Expert (V.22.0.6). In the present work, Box-Behnken design (BBD) was employed with three numerical factors (GLDA concentration, pH, and retention time) at three levels. These factors were examined as independent variables. BBD and RSM can be used in continuous range analysis, with the short experimental cycle, and the advantages of high precision. The primary objective of employing Response Surface Methodology (RSM) is to ascertain the optimal operational parameters for the system that meets the operational specifications. The minimum and maximum range selected for the RSM was based on the equilibrium conditions determined using the one-variable-at-a-time approach. This resulted in 15 experimental runs, including 12 runs for factorial design points and 3 runs for identical design points at the centre. The allowed values for the independent variables were [− 1], [0], and [1]. All the experiments were performed in triplicate to obtain average data of metals recovery (Cd, Cu, Pb and Zn). Design expert was utilized to perform the statistical analysis of the experimental data and received the regression mode. By comparing the predicted values of the model with the experimental values to obtain the highest degree of fit of the model. Figure 5 provides a description of the RSM optimization technique employed in this investigation. The model for determining the optimum combination and understanding the relationship between the specified variables is expressed in the form of a generalized quadratic equation (Eq. (1)):

1 $$R={\gamma }_{0}+\sum_{i=1}^{n}{\gamma }_{i}{k}_{i}+\sum_{i=1}^{n}{\gamma }_{ii}{k}_{i}^{2}+\sum_{i=1}^{n-1}\sum_{j=i+1}^{n}{\gamma }_{ij}{k}_{i}{k}_{j}$$

where, R represents the expected/projected outcome, γ_0_ is the constant term in the equation, γ_i_ denotes the linearity coefficient, γ_j_ relates to the quadratic coefficient and γ_ij_ signifies interactive coefficient. The coded variables k_i_ and k_j_ correspond accordingly while n stands for individual variables.

### Sequential extraction experiment

Geochemical fractions of Cd, Cu, Pb, and Zn in MSW compost samples pre- and post-treatment were extracted step by step using a slightly modified version of Tessier's sequential extraction procedure (TSEP)^19,21^. The process is illustrated in Fig. S1. Metal fractions are grouped into six categories, designated as F1, F2, F3, F4, F5, and F6 as shown in Fig. S2. Additionally, the sequential extraction efficiency [SEE (%)] was computed by the ratio of the sum of all fractions to the total concentration.

### Analysis of data

The reported mean values include error bars representing the standard deviations of three distinct samples. Analysis of variance and regression on the experimental data were conducted using Design Expert (V.22.0.6), and Minitab (V.19.1). Additionally, kinetic models such as pseudo-first order and pseudo-second order models were applied to analyze the kinetic data for fitting purposes. The optimal kinetic model selection was based on calculating the coefficient of determination (R^2^). To differentiate means at a significance level of *p* < 0.05, Fisher’s least significant difference test was utilized.

### Consent for publication

All the authors have their consent for publishing the manuscript.

## Result and discussion

### Physiochemical properties of original MSW compost

The physicochemical analysis of MSW compost is crucial for understanding its characteristics, which in turn affect metal pollution and the development of remediation plans. Table 2 provides an overview of the physicochemical properties and heavy metal levels in MSW compost. The pH was measured at 6.4, indicating a slightly acidic nature that can enhance the mobility and bioavailability of heavy metals^53^. Meanwhile, the EC value of 2.2 ds/m suggests low salinity levels. With a bulk density of 0.64 g/cm^3^ and relatively low organic content, these values indicate both organic matter and inert material/ash content on a dry weight basis. Composts with lower bulk density are preferred as they increase water retention when applied over extended periods^54^.

Organic material has the ability to bind with heavy metals, influencing their movement and availability. The metal levels in the original MSW compost sample show concentrations of 21.80, 1836.51, 698.35, and 8130.73 mg/kg (dry mass basis) for Cd, Cu, Pb, and Zn respectively. These levels surpass the environmental quality standard for compost (IS16556:2016)^52^, indicating unsuitability for agricultural use. This non-compliance could result in significant heavy metal contamination with adverse effects on the environment. The main reason behind elevated metal content in the initial MSW compost is the presence of Cd, Cu, Pb, and Zn commonly found in paints; electrical components; batteries; ceramics; fungicides; electroplating among others. These metals accumulate in municipal solid waste before being transferred to municipal solid waste compost through composting processes as they are inorganic and non-biodegradable^42^. More extensive findings for metal levels from microstructural examination are outlined in our earlier publication by Khare et al.^12^. The MSW compost has a particle size of ‘ < 4 mm’, leading to increased permeability, which may enhance the leaching of heavy metals and therefore potentially deteriorate the risk of groundwater contamination. The complex interplay between these conflicting interactions ultimately dictates the overall efficiency of removing heavy metals from municipal solid waste compost.

### GLDA-assisted leaching of heavy metal contaminated MSW compost

#### GLDA as high potential complexing agent

Depending on the pH of the solution, GLDA molecules undergo dissociation. The phases of dissociation correspond to specific protonation constants. In strong acidic solutions (pH = 2), GLDA exists as a fully protonated molecule (H_4_GLDA). As pH increases, protons progressively detach from the oxygen atoms in the carboxyl groups, following successive logK values (stability constant) and leading to deprotonation in the liquid phase at equilibrium state^28^ (Eqs. (2)–(5)). Conversely^48^, under highly basic conditions, the last dissociation occurs with the proton from the amine nitrogen atom, resulting in a fully deprotonated form of GLDA^4^.

2 $$ {\text{H}}_{{4}} {\text{GLDA }} \leftrightarrow {\text{ H}}_{{3}} {\text{GLDA}}^{ - } + {\text{ H}}^{ + } $$

3 $$ {\text{H}}_{{3}} {\text{GLDA}}^{ - } \leftrightarrow {\text{ H}}_{{2}} {\text{GLDA}}^{{{2} - }} + {\text{ H}}^{ + } $$

4 $$ {\text{H}}_{{2}} {\text{GLDA}}^{{{2} - }} \leftrightarrow {\text{ H}}_{{3}} {\text{GLDA}}^{{{3} - }} + {\text{ H}}^{ + } $$

5 $$ {\text{HGLDA}}^{{{3} - }} \leftrightarrow {\text{ GLDA}}^{{{4} - }} + {\text{ H}}^{ + } $$

In this study, the recommended pH was 2.9 for GLDA, indicates that the compound exists^48^ in the form of H_3_GLDA^-^. Consequently, GLDA undergoes protonation or deprotonation as the pH decreases or increases. Protons obstruct the metal binding sites in MSW compost, causing metals initially fixed in the bimolecular layer of MSW compost colloid to be released into the liquid phase in their ionic form^55^. The balance of species in a metal–ligand system depends on the concentrations of all metals and ligands, as well as the stability constants of all complexes. When another metal–ligand complex or metal ion is introduced to the solution, a new equilibrium will be established. The coordination reactions between metal ions and ligands containing multiple bonding groups are often slowed down kinetically. Several variables can impact the stability of metal complexes, including the central metal ion's nature, ligand properties, chelating effect, macrocyclic effect, resonance effect, and steric hindrance^56^. The stability constant represents the increased stability of these complexes; higher values indicate greater stability^47^. According to research findings^47,55^, the observed values for the stability constants are as follows: Cu (13.03) > Pb (11.60) > Zn (11.52) > Cd (10.31). Interestingly, this research discovered a different trend in removal efficiency: Cd > Zn > Cu > Pb—contradicting the previously reported sequence. Pinto et al.^28^ proposed that this variation could be attributed to factors such as the disintegration rate of metal complexes and metal oxides on MSW compost surfaces.

#### Process conditions for GLDA-assisted leaching of heavy metals

**Figure 6:**
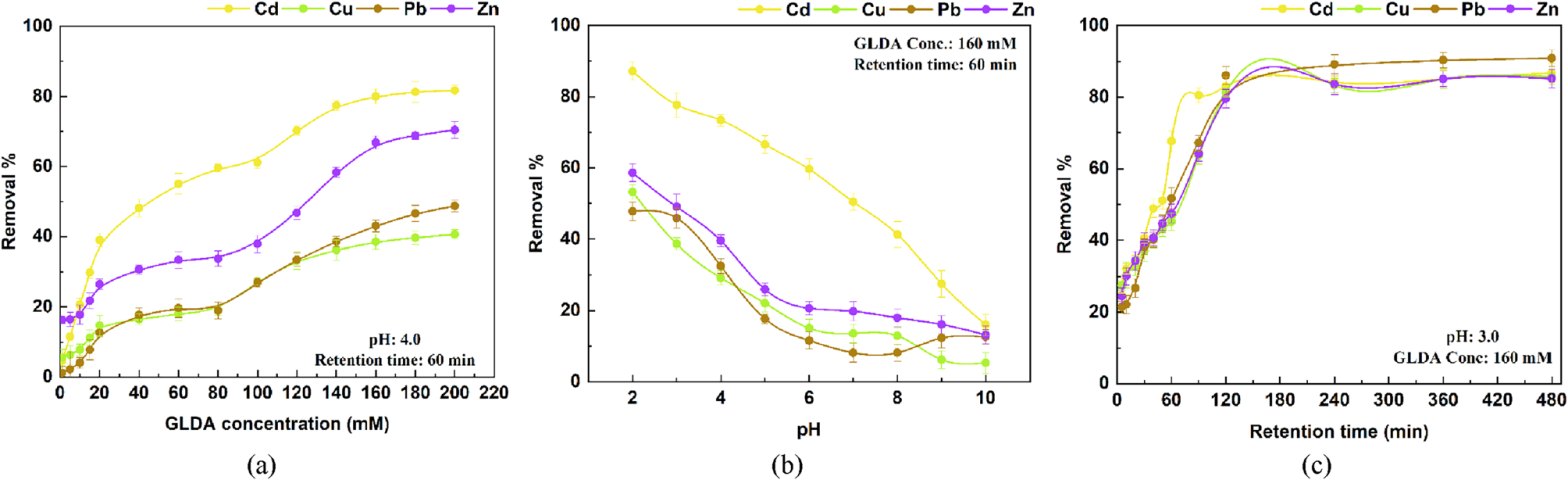
Leaching effect of (**a**) GLDA concentration, (**b**) pH, and (**c**) retention time on removal efficiencies of Cd, Cu, Pb, and Zn from MSW compost, respectively. Values reflect the mean ± standard deviation of three trials.

***Leaching effect on GLDA concentration***: Chelator concentration is a crucial element that impacts the effectiveness of metal extraction by chelators in the process of leaching remediation for contaminated MSW compost^43^. Consequently, the impact of chelator concentration on metal extraction was investigated by combining MSW compost with different levels of GLDA ranging from 1 to 200 mM. Table S1 presents the leaching effect of GLDA concentration on the removal efficiencies of Cd, Cu, Pb and Zn from MSW compost. The highest removal percentages for Cd, Cu, Pb, and Zn were 81.7%, 40.6%, 48.6%, and 70.4% after leaching with GLDA. These findings were also aligned with a study carried out by Wu et al.^57^ which documented similar results. In the presence of GLDA, the study noted approximately 82% removal of Cd at pH 4.0. Meanwhile, removal of Zn remained persist. According to Suanon et al.^15^, the variations in results may be explained by changes in the heavy metal concentration within the waste and underlying circumstances in the environment, including toxic metals’ chemical composition and the existence of non-target elements. Figure 6a indicates that the removal efficiencies of Cd, Cu, Pb, and Zn exhibited a sharp increase with increasing GLDA concentrations up to 140 mM. The gains were gradual after 140 mM until 160 mM. This observation is in line with previous studies showing that higher chelator concentrations enhance the solubilization of heavy metals^16,47^. The increased availability of chelators for complexation with metal ions can be attributed to the increase in extraction efficiency, facilitating their mobilization from the MSW compost matrix^58^. An elevated concentration of chelating agent leads to increased binding of metal ions that are toxic which facilitates metal ion–ligand complexes formation, thus enhancing removal efficiency. This effect is attributed to the availability of more effective binding sites due to the higher concentration of the chelating agent^16,55^. Recent research discovered that GLDA exhibits stronger metal-binding capabilities at lower concentrations due to its higher number of functional groups and forms stable water-soluble complexes with metal ions^59^. However, the concurrent dissolution of target metals and other elements such as Fe, Mg, Al and Ca may reduce the overall removal efficiency, especially when the chelating agent concentration is low^60^.

On the other hand, at GLDA concentrations above 160 mM, there were no significant improvements in removing these metals. Higher concentrations of GLDA did not contribute towards enhanced removal of Cd, Cu, Pb, and Zn in MSW compost. This may be the result of reaching full capacity of metal-binding sites after rinsing with increased chelator concentrations. A previous study has also observed a comparable occurrence^21,30^. As a result, a range from 130 to 170 mM was chosen for additional statistical refinement in determining the optimal chelator concentration for GLDA-assisted leaching of metal-contaminated MSW compost.

***Leaching effect on pH*:** The pH of the leaching solution plays a significant role in the effectiveness of chelators during the GLDA-assisted leaching of metal contaminated MSW compost. The pH level of the solution is believed to impact the solubility of heavy metals, as well as the structure and charge of chelators, and their interaction with metal ions^28^. As a result, contaminated MSW compost underwent treatment with GLDA solutions at different pH levels (ranging from 2 to 10) in order to study how the solution's pH affected the removal of Cd, Cu, Pb, and Zn with GLDA-assisted leaching. Table S2 provides leaching effect of pH on the removal efficiencies of Cd, Cu, Pb and Zn from MSW compost. The highest removal efficiencies were achieved at very low pH of 2, with 87.1%, 53.2%, 47.7%, and 58.6% for Cd, Cu, Pb, and Zn, respectively. Whereas at pH 4, the removal efficiencies were 73.3%, 29.2%, 32.5%, and 39.5% for Cd, Cu, Pb, and Zn, respectively (Fig. 6b). This observation is consistent with the fact that reduced pH levels promote metal dissolution and subsequent binding with chelating agents^61^. The acidic conditions facilitated the dissolution of metals bound to the MSW compost, causing them to readily dissolve in the lower pH environment^50^. The affinity of metals for a ligand plays a crucial role in forming metal–ligand complexes, where R–(COOH)m is an organic reagent (leaching solution) and Mn^+^ represents a metallic ion. The molecular structure of GLDA, with its four carboxyl groups (Fig. S1), explains this observation. According to Zaleckas et al.^62^, a greater capacity for metal extraction is exhibited by a molecule with more carboxyl groups compared to one with fewer carboxyl groups. Consequently, the removal efficiencies for toxic metals were higher at lower pH conditions. A decrease in the removal of Cd, Cu, Pb, and Zn was noted for GLDA as the pH of the solution increased from 3 to 10. The lowest efficiency in extracting metals was observed at pH 7. This pattern could be explained by increased competition from protons (H^+^) at lower pH levels and the formation of metal-hydroxide precipitates at higher pH levels, leading to a decrease in metal oxide solubility^47^. Complexation between heavy metals and chelators can be disrupted by these factors. The overall binding strength of the chelating agent may decrease as the pH increases. Consequently, the ideal pH for removing heavy metals seems to differ based on the particular chelator utilized^45^. Additionally, the leaching treatment at low pH can impact the fertility characteristics of MSW compost^50^. Therefore, it is advisable to allow saturated MSW compost after leaching to undergo open drying for 48 h in order to promote sufficient air circulation. This aeration process creates turbulence, leading to the release of aqueous CO_2_ (carbon dioxide), which reduces acidity and raises the pH level^63,64^. Furthermore, natural additives such as agricultural lime, crushed eggshells, bone meal, and calcium hydroxide can be utilized or bioaugmentation or cultivation could also be employed to slightly increase the pH level and enhance attributes of treated MSW compost. Moreover, a range of 2.5–3.5 for pH levels is further fine-tuned using statistical optimization through a RSM model.

***Leaching effect on retention time*:** Retention time plays a crucial role in the chelator-assisted removal of heavy metals from contaminated MSW compost. It directly affects the duration of interaction between the MSW compost and the chelator solution^43^. To understand its impact, the extraction of Cd, Cu, Pb, and Zn using GLDA was studied at different retention times (0, 10, 20, 30,40, 50, 60, 90, 120, 240, 360, 480 min) while maintaining constant pH. Table S3 shows leaching effect of retention time on the removal efficiencies of Cd, Cu, Pb and Zn from MSW compost. In this study, the removal efficiencies of Cd, Cu, Pb, and Zn showed a significant increase within the first 60 min of leaching, with values of 67.6%, 45.1%, 51.7%, and 64.1% respectively (*p* < 0.05). Subsequently, the removal efficiencies continued to rise at a slower rate, reaching equilibrium at around 120 min with removal efficiencies of 83.0%, 81.3%, 86.0%, and 79.5% for Cd, Cu, Pb, and Zn, respectively (Fig. 6c). A prominent linear relation (*p* < 0.05) was established between removal efficiencies and retention time. As the leaching process extended beyond 120 min, the removal efficiencies remained relatively constant, even up to a retention time of 480 min. The findings indicate that the leaching of Cd, Cu, Pb, and Zn from MSW compost using GLDA involves a two-phase kinetic process. The first phase, which occurs swiftly within the initial 60 min, is succeeded by a steady extraction process over the resulting hours.

This pattern aligns with previous research examining the soil washing time^65^. The phenomenon can be attributed to the movement of metals that are weakly attached can easily form complexes with GLDA and are rapidly released, leading to a sharp increase in metal removal efficiencies^31^. As the contact period increases, GLDA forms complexes with stable metal species, progressively improving the metal removal efficiency at a slower rate. Therefore, for achieving optimal conditions a range from 120 to 180 min of retention time was selected for statistical analysis.

In this research, the kinetic parameters were also evaluated using the pseudo-first and second order rate equations (Equations S1 and S2, respectively). The fitting of these two kinetic models to the equilibrium data of toxic metals is presented in Fig. S3. The pseudo-second-order kinetic model provides the best fit for describing the leaching kinetics of toxic metals in MSW compost. This model exhibited higher values of the linear regression coefficient (R^2 ^> 0.99, in all cases) compared to the pseudo-first-order model. These findings suggest that when biodegradable chelator is used for remediation of Cd, Pb, and Zn polluted soil, the chemisorption-controlled mechanism is involved during desorption process^65^, which entails valence forces resulting from electron transfer between the functional groups in the washing solutions and the heavy metal ions. This observation confirms that metal ions can bond with the functional groups in the washing solutions. The results of this study align with those of previous research, such as the works of^31^, where the pseudo-second-order model was utilized to remediate soil contaminated with Cd, Pb, and Zn.

Furthermore, the higher k_2_ value, indicating stronger mobility, was found for Pb > Cd > Cu > Zn in MSW compost, influencing the metal removal efficiency. These findings align with a previous investigation consistent with study conducted by Kulikowska et al.^53^, which displays a higher removal of Cd compared to Pb due to the presence of humic compounds. The presence of humic compound in MSW compost can both enhance and hinder metal chelation^66,67^. It enhances chelation through complexation and the production of organic ligands as it contains diverse functional groups such as carboxyl, hydroxyl, and phenolic groups that can form stable metal-complexes and increases metal solubility. Additionally, organic matter can produce natural chelating agents like fulvic and humic acids which bind strongly with metal ions, preventing their precipitation and facilitating their transport within the compost^68^. However, it hinders chelation by strong sorption to stable organic fractions of organic matter, particularly in humus-rich regions of the compost, formation of insoluble complexes, and competitive binding sites due to high level of organic matter content, reducing metal availability for biological uptake^68,69^. The results were also aligned with previous research investigating the process of kinetic equilibrium^31^. According to Tang et al.^70^, the extraction process taking retention time into account, can be elucidated through two mechanisms: a gradual phase involving external diffusion, robust dissolution, and thorough scrubbing, resulting in peak extraction efficiency. Equilibrium concentration was reached within 120 min of extraction for all metals. The observation is aligned to the fact that k_2_ values are primarily influenced within the initial minutes of the leaching process, as noted by Kulikowska et al.^53^. Hence, to achieve equilibrium conditions retention time may have greater importance than k_2_ value.

#### Features of GLDA-assisted leaching mechanism

When managing MSW compost contaminated with heavy metals using GLDA-assisted leaching, the chelation process encompasses multiple stages such as protonation/deprotonation equilibria, interactions between metal ions and ligands, and reactions occurring at the interface of solid and liquid phases. The specific mechanism derived from these findings is provided below:

The initial stage of utilizing MSW compost to eliminate heavy metals begins with the liberation of unbound H + ions from GLDA, a polydentate chelating agent. This facilitates the breakdown of alkaline oxides and enhances the solubilization of metals. The equilibrium reactions^28,47^ for different forms of GLDA are illustrated in Eqs. (2)–(5). Upon decreasing or increasing the pH, the GLDA could be either protonated or deprotonated^46^. The existence of protons disturbs the binding sites of heavy metals in MSW compost, leading to the release of heavy metals that were originally trapped in the biolayer of compost colloids into the liquid phase in an ionic state^55^.

Moreover, a comprehensive understanding of GLDA's capacity for heavy metal removal involves considering the interaction between metal ions and complexation. The interaction of GLDA (L^-^) and heavy metal ions (M^2+^) leads to the formation of reversible metal–ligand complexes at a 1:1 ratio^47,57^. These interactions are described by reactions Eqs. (6)–(9):

6 $$ {\text{M}}^{{{2} + }} + {\text{ H}}_{{3}} \left( {\text{L}} \right)^{ - } \leftrightarrow \, \left[ {{\text{MH}}_{{3}} \left( {\text{L}} \right)} \right]^{ + } $$

7 $$ {\text{M}}^{{{2} + }} + {\text{ H}}_{{2}} \left( {\text{L}} \right)^{{{2} - }} \leftrightarrow \, \left[ {{\text{MH}}_{{2}} \left( {\text{L}} \right)} \right] $$

8 $$ {\text{M}}^{{{2} + }} + {\text{ H}}\left( {\text{L}} \right)^{{{3} - }} \leftrightarrow \, \left[ {{\text{MH}}\left( {\text{L}} \right)} \right]^{ - } $$

9 $$ {\text{M}}^{{{2} + }} + \, \left( {\text{L}} \right)^{{{4} - }} \leftrightarrow \, \left[ {{\text{M}}\left( {\text{L}} \right)} \right]^{{{2} - }} $$

Due to the formation of metal–ligand complexes (M–L), the stability constant (log K) of the complex is defined to represent its thermodynamic stability, with higher log K values indicating greater stability. However, in our study, we noted the log K values (in ascending sequence) for Cu, Pb, Zn, and Cd as 13.03, 11.60, 11.52, and 10.31 correspondingly^47,55^. Whereas we found a different pattern of removal efficiency: Cd > Zn > Cu > Pb. This contradicts the previously suggested sequence. According to Pinto et al.^28^ the differences in variation might result from factors like the breakdown rate of metal complexes and metal oxides on the surface of MSW compost.

The GLDA^-^assisted leaching can be categorized into two stages, depending on the target heavy metals (M_i_^m+^) and other constituents found in the MSW compost matrix. The initial stage involves the interplay between the metal and GLDA (L^n−^, n = 0, 1, 2, 3, 4) present in the solution at the interface of solid–liquid phases. The interaction between GLDA and heavy metals involved incorporating metal ions into the heterocyclic structures of GLDA, replacing monodentate ligands with multidentate ligands to form M–L complexes Ferraro et al.^55^. The substitution processes show that the GLDA replaces other components (e.g., OH^−^ ions) in the MSW compost (Compost) matrix to form metal-GLDA complexes. Formation of these complexes aids in extracting metals from solid matrix into liquid phase. This includes reactions Eqs. (10)–(12) such as:

10 $$ \left( {{\text{Compost }}{-}{\text{ O}}} \right) \, {-}{\text{ M}}_{{\text{i}}}^{{{\text{m}} + }} + {\text{ L}}^{{{\text{n}} - }} + {\text{ H}}_{{2}} {\text{O }} \leftrightarrow \, \left( {{\text{Compost}}} \right) \, {-}{\text{ OH }} + {\text{ M}}_{{\text{i}}} {-}{\text{ L}}^{{{\text{m}} + \left( {{\text{n}} - {1}} \right) - }} + {\text{ OH}}^{ - } $$

11 $$ \left( {{\text{OH}}} \right)_{{\text{m}}} {\text{M}}_{{\text{i}}} + {\text{ L}}^{{{\text{n}} - }} \leftrightarrow {\text{ M}}_{{\text{i}}} {-}{\text{ L}}^{{{\text{m}} + {\text{n}} - }} + {\text{ mOH}}^{ - } $$

12 $$ {\text{M}}_{{\text{i}}} {\text{O }}\left( {{\text{M}}_{{\text{i}}} {\text{O}}} \right)^{{{\text{m}} + {2} - }} + {\text{ L}}^{{{\text{n}} - }} + {\text{ H}}_{{2}} {\text{O }} \leftrightarrow {\text{ M}}_{{\text{i}}} {-}{\text{ L}}^{{{\text{m}} + {\text{n}} - }} + {\text{ 2OH}}^{ - } $$

In the second stage, two primary processes occur: surface complexation (Eq. 13) or ion-exchange of metals with other cationic species (M_j_^mj+^) in solution like K^+^ and Ca^2+^ ions (Eqs. 14, 15); and the second process suggests that some metals may not form complexes or undergo adsorption but remain in a free state in the liquid phase (Eq. 16).

13 $$ \left( {\text{Compost}} \right) \, {-}{\text{OH}} +{\text{M}}_{\text{i}}^{\text{m}+} {-}{\text{L}}^{{\text{n}} - }\leftrightarrow \, \left( {\text{Compost}} \right) \,{-}{\text{L}}^{{\left( {{\text{n}} - {1}} \right) - }} {-}{\text{M}}_{\text{j}}^{\text{m}+} +{\text{OH}}^{-} $$

14 $$ \left( {\text{Compost }} \right) \, {-}{\text{ L}}^{{{\text{n}} - }} {-}{\text{ M}}_{{\text{i}}}^{{{\text{mi}} + }} + \, \left( {{\text{Compost}}} \right) \, {-}{\text{ O }}{-}{\text{ M}}_{{\text{j}}}^{{{\text{mj}} + }} + {\text{ H}}^{ + } \leftrightarrow \, \left( {{\text{Compost}}} \right) \, {-}{\text{ L}}^{{{\text{n}} - }} {-}{\text{ M}}_{{\text{j}}}^{{{\text{mi}} + }} + \, \left( {{\text{Compost}}} \right) \, {-}{\text{ OH}}^{ + } + {\text{ M}}_{{\text{i}}}^{{{\text{mj}} + }} $$

15 $$ \left( {{\text{Compost}}} \right) \, {-}{\text{ L}}^{{{\text{n}} - }} {-}{\text{ M}}_{{\text{j}}}^{{{\text{mi}} + }} + {\text{ OH}}^{ + } \leftrightarrow \, \left( {{\text{Compost}}} \right) \, {-}{\text{ OH}}^{ + } + {\text{ M}}_{{\text{j}}}^{{}} {-}{\text{ L}}^{{{\text{m}} + \left( {{\text{n}} - {1}} \right) - }} $$

16 $$ \left( {{\text{Compost}}} \right) \, {-}{\text{ OH }} + {\text{ M}}_{{\text{i}}}^{{{\text{m}} + }} \leftrightarrow \, \{ \left( {{\text{Compost}}} \right) \, {-}{\text{ O}}]{\text{ M}}_{{\text{i}}}^{{{\text{m}} + {1} - }} + {\text{ H}}^{ + } $$

Based on these mechanisms, it is implied that several processes are involved in the elimination of heavy metals through GLDA-assisted leaching:

- Solubilization Effect: Metal oxides can solubilize, releasing metals into the solution.
- Stable Metal-GLDA Complexes: formation of stable M–L complexes by sorption with compost organic matter function groups and linked to form organic matter/metal hydroxides
- Ion exchange: Heavy metals undergo surface binfding, with K^+^ by electrostatic outer–sphere complexation and exchange with Ca^2+^ by inner-sphere complexation and co-precipitation
- Surface complexation: Heavy metals engage in surface binding with available complexing sites on GLDA.

Overall, these processes help in effectively eliminating heavy metals from MSW compost, suggesting that GLDA-assisted leaching could be a powerful technique for addressing contaminated MSW compost. It is also important to note that, to manage chelated leachates with heavy metals and prevent secondary pollution after biodegradation, leachates are treated using methods like chemical precipitation, ion exchange, adsorption, or membrane filtration. Additionally, biological treatments such as bioremediation with microorganisms or phytoremediation with plants can be employed. Of course, regular monitoring of treated leachates ensures that metal concentrations meet environmental safety standards and that no harmful byproducts are present. Table S4 outlines effective techniques for converting chelated metal-complex solutions into useful resources, offering valuable support for both environmental decontamination and addressing scarcity of resources.

### Optimal conditions obtained using Response surface method

The process conditions for GLDA-assisted leaching of heavy metals from MSW compost were optimized using a three-level, three-factor Box-Behnken design (BBD) in RSM. BBD experimental range and level of independent variables are provided in Table 3. The design of experiment proposed by BBD and the results are presented in Table S5. A polynomial model was utilized to analyze the data displayed in Table S6, which relates to the efficiency of metal removal. The impact of each factor on the model's response was investigated using analysis of variance. The experimental data was analyzed through multiple regression to fit the results of the BBD to a modified quadratic polynomial equation. The final models in terms of coded factors for the leaching rates of Cd, Cu, Pb, and Zn are provided in Eqs. (17)–(20), respectively. These equations represent k_1_, k_2_, k_3_ as GLDA concentration, pH, retention time; while interaction factors are represented by k_1_k_2_, k_1_k_3_, and k_2_k_3_ are the interaction factors.

**Table 3:** BBD experimental range and level of independent variables.

Process variables	Code	Level range	
		− 1	+ 1
GLDA concentration (mM)	*k*_*1*_	130	170
pH	*k*_*2*_	2.5	3.5
Retention time (min)	*k*_*3*_	120	180

17 $${R}_{Cd}=87.22+2.21{k}_{1}-1.59{k}_{2}-0.30{k}_{3}+0.52{k}_{1}{k}_{2}-0.89{k}_{1}{k}_{3}-0.40{k}_{2}{k}_{3}-0.65{k}_{1}^{2}-0.14{k}_{2}^{2}+0.25{k}_{3}^{2}$$

18 $${R}_{Cu}=36.50+1.58{k}_{1}-1.95{k}_{2}-17.97{k}_{3}+1.58{k}_{1}{k}_{2}-0.33{k}_{1}{k}_{3}-2.92{k}_{2}{k}_{3}+1.80{k}_{1}^{2}+2.29{k}_{2}^{2}+17.13{k}_{3}^{2}$$

19 $${R}_{Pb}=42.53+1.65{k}_{1}-1.30{k}_{2}-18.02{k}_{3}+2.41{k}_{1}{k}_{2}+0.12{k}_{1}{k}_{3}-1.55{k}_{2}{k}_{3}+0.95{k}_{1}^{2}-1.18{k}_{2}^{2}+18.04{k}_{3}^{2}$$

20 $${R}_{Cd}=56.67+4.61{k}_{1}-0.75{k}_{2}-10.23{k}_{3}+1.61{k}_{1}{k}_{2}+3.22{k}_{1}{k}_{3}+0.88{k}_{2}{k}_{3}-1.14{k}_{1}^{2}+0.27{k}_{2}^{2}+10.18{k}_{3}^{2}$$

According to Table S6, models with F-values of 24.56, 128.08, 389.8, and 41.78 (*p* < 0.05) demonstrate significant statistical relevance. Moreover, the conformity between projected and experimental values for MSW compost is apparent from the high R^2^ and R^2^ adj. values close to 1.0, indicating substantial explanatory power in the models. The experiments were also highly reliable and precise with a coefficient of variation of *p* < 0.05 for all metals^71^. The study additionally evaluated the model's precision (adequate precision) using signal-to-noise ratio^31^. The precision values of 18.09, 28.61, 51.29, and 19.45 indicate that all four models were satisfactory. The linear effects of GLDA concentration, pH, and retention time exhibited statistical significance based on results obtained from significance tests.

The experimental data illustrated in Fig. S4 demonstrate adherence to a normal distribution, indicating that the data maintained their regularity without any alteration of responses. The effectiveness of the constructed models was confirmed through ANOVA and diagnostic plots, with statistical significance (*p* < 0.05) and non-symmetrical scattering in residual versus projected value plots (Fig. S5 b, d, f, h). Additionally, it was found that quadratic models effectively controlled heavy metal removal based on similar distributions of projected removal efficiencies for Cd, Cu, Pb, and Zn from both models and observed experimental data along the 45° line (Fig. S5 a, c, e, g). These results indicate that the BBD model accurately optimizes toxic metal removal.

#### Effect of 3D contour interaction

**Figure 7:**
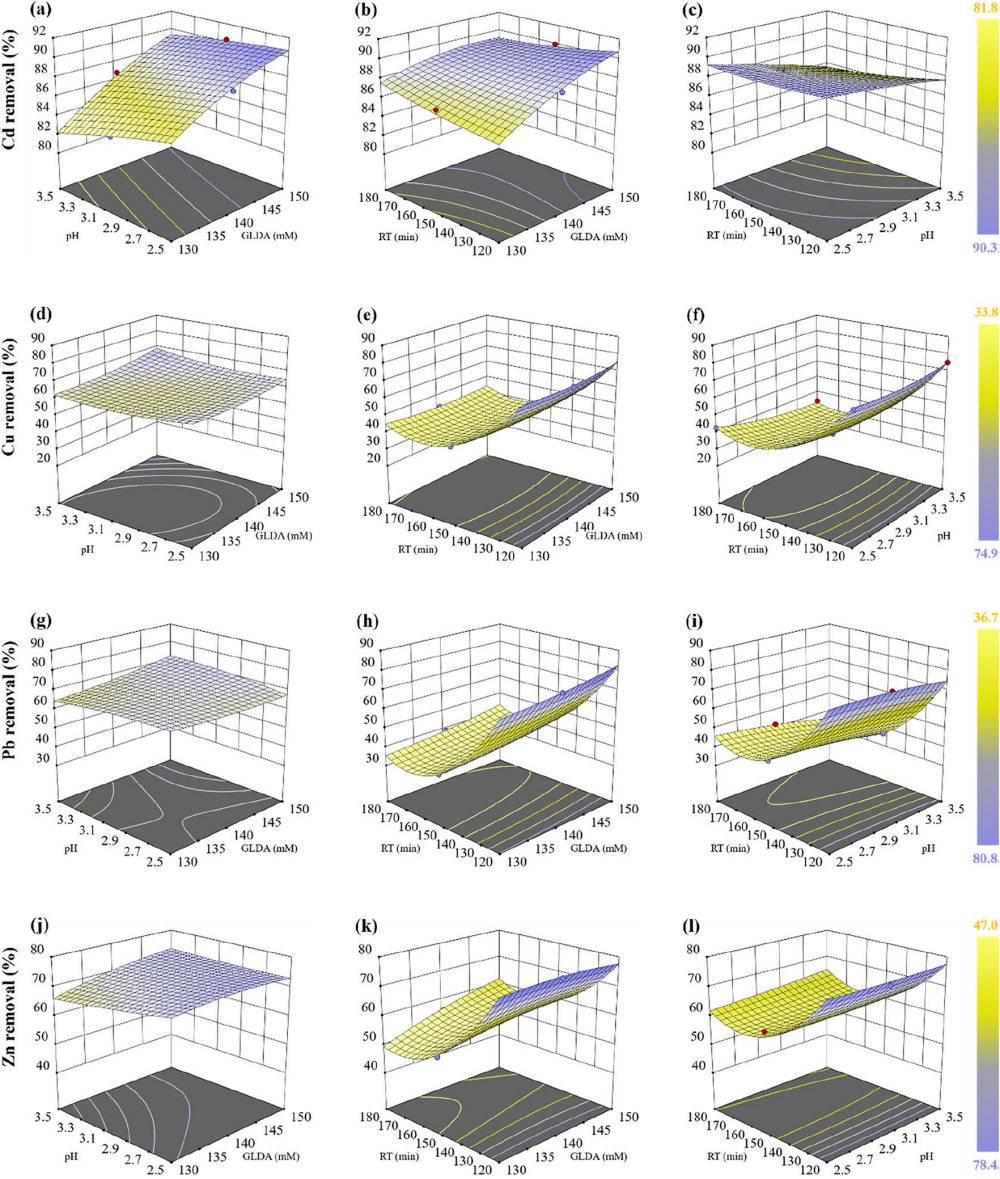
3D plots illustrating the relationship between two distinct variables and their impact on removal efficiencies of Cd (**a**, **b**, **c**); Cu (**d**, **e**, **f**); Pb (**g**, **h**, **i**); and Zn (**j**, **k**, **l**).

RSM was employed to analyze the interaction effects of two independent factors on the leaching of Cd, Cu, Pb, and Zn. Contour plots were generated based on the fitted model equations to illustrate these effects. Figure 6 presents contour plots depicting the impact of variables on the leaching percentage of Cd (Fig. 7a, b, c), Cu (Fig. 7d, e, f), Pb (Fig. 7 g, h, i, and Zn (Fig. 7j, k, l). The contour lines directly indicate the degree of interaction between two factors; a circle signifies that there is no significant interaction whereas an ellipse indicates a significant interaction^65^. Moreover, Tindanzor et al.^72^ highlighted that the center of an elliptical representation represents optimum conditions. In terms of single variable impact, GLDA concentration, pH, and retention time exhibited distinct influences on leaching rates of target metals.

As GLDA concentration climbed from 130 to 150 mM, the removal efficiencies of Cd, Cu, Pb, and Zn in MSW compost increased continuously. However, at lower pH conditions the impact was more significant. This observation aligns with the results reported in previous research^50,57^. The metal removal efficiency gradually decreased to 120 min of retention time, then exhibited an upward trend in response to greater concentrations of GLDA. The highest metal removal efficiencies were obtained on using a concentration of 170 mM GLDA and a retention time of 120 min, with the exception of Zn, which required a concentration of 150 mM GLDA and the same retention time. An increase in the heating agent's concentration and retention time duration may facilitate the disruption of the metals' chemical bonds in MSW compost, leading to more effective metal removal^60^. The elimination of Cd, Cu, Pb, and Zn was also affected by the relation between pH and retention time, as shown by the curved surface variation. The removal efficiencies of heavy metals would improve at lower pH and shorter retention time, indicating that an increase in hydrogen ions (H^+^) causes a significant release of metals in MSW compost via ion exchange, which increases protonation of the surface colloids^31^. The results of the three-factor design method agreed with this finding.

#### Optimum conditions

The optimization of parameters using RSM indicates that GLDA-assisted leaching of MSW compost is observed at GLDA concentration of 150 mM, pH value of 2.9, and a retention time of 120 min. At these optimal conditions, the anticipated removal efficiencies for Cd-90.32%, Cu-81.96%, Pb-91.62%, and Zn-80.34%. The validation test results show values of 90.07% for Cd, 80.44% for Cu, 92.28% for Pb, and 81.95% for Zn at 95% confidence and prediction intervals. So, the models are well fitted and highly significant. Thus, the findings suggest that BBD is a valuable method for enhancing the toxic metal removal efficiency.

#### Assessing metal extraction efficiency: comparison of EDTA and GLDA-assisted leaching at optimal conditions

A comparison experiment was performed to assess Cd, Cu, Pb, and Zn removal effectiveness using GLDA and conventional chelating agent EDTA under optimal operational conditions (150 mM GLDA concentration, 2.9 pH, and 120 min retention time). GLDA-assisted leaching obtained the removal efficiency of Cd as 90.07%, Cu as 80.44%, Pb as 92.28%, Zn as 81.95%. The findings indicated that EDTA demonstrated excellent extraction efficiency for Cd (94.08%), Cu (82.36%), Pb (93.16%) and Zn (85.74%) in comparison to GLDA, as outlined in Table 2. This superior performance of EDTA is because of the presence of carboxylic acid groups in high concentration and also results in more stable metal complexes, as discussed by Begum et al.^46^. However, it is worth noting that GLDA also demonstrated substantial removal of Cd, Cu, Pb, and Zn, although not as efficient as EDTA. Despite the effective extraction capabilities of EDTA, its use poses environmental risks due to its non-biodegradability and potential adverse effects on ecosystems, such as promoting eutrophication, as mentioned in prior research^32^. As highlighted in a study^21^, GLDA is biodegradable and non-toxic to the ecosphere. Therefore, GLDA is a practical and eco-friendly chelating agent for addressing severe metal contamination in MSW compost. By employing GLDA technology, not only environmental impact of heavy metal pollution is minimized but it also promotes the reuse and recycling of resources, contributing towards the circular economy. The quality and classification of the GLDA-treated MSW compost can be determined using the criteria outlined by Saha et al.^54^, which includes marketable classes (A, B, C, D) or restricted use classes. This classification will define the specific purpose and application of the compost accordingly. Regarding the potential reuse of GLDA-treated MSW, a thorough research by Saha et al.^54^ proposes a scheme for categorizing composts into different marketable classes (A, B, C, and D) and restricted use classes (RU-1, RU-2, and RU-3) based on their fertilizing potential and potential for contaminating the soil and food chain, which is beneficial for relevant stakeholders (Table S7).

### Understanding geochemical fractionation in MSW compost: pre- and post-treatment analysis

**Figure 8:**
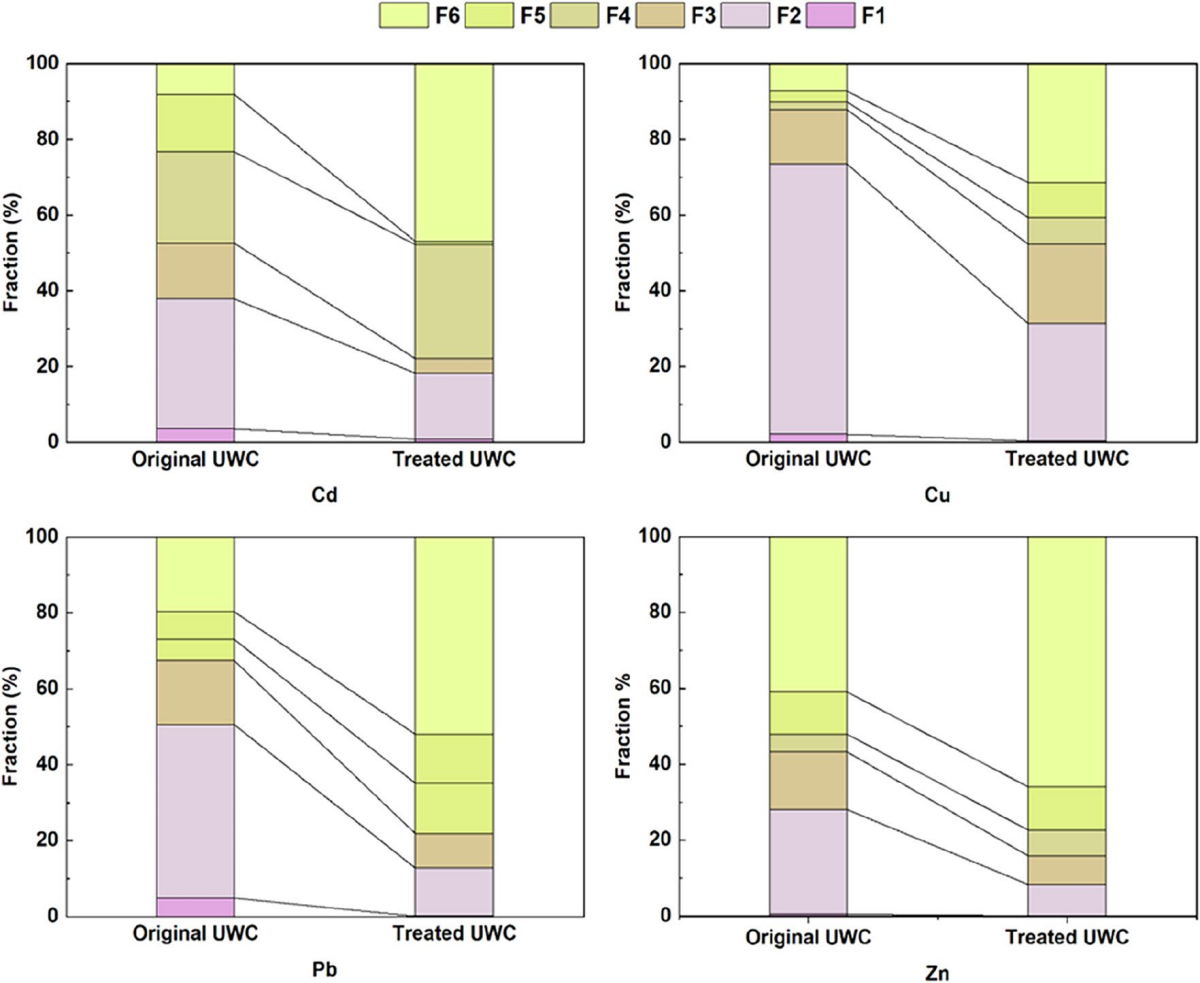
Geochemical distribution of Cd, Cu, Pb, and Zn in original and treated MSW compost (at optimum condition: 150 mM, pH 2.9, and retention time 120 min).

The geochemical distribution of metals in various fractions within MSW compost is essential for comprehending the affinity between metal pollutants, their mobility, bioavailability, and detailed information regarding pollutant toxicity. Therefore, a sequential extraction was carried out on pre- and post-treated MSW compost to examine the geochemical distribution of Cd, Cu, Pb, and Zn across different phases as discussed in Section "Sequential Extraction Experiment". Table S8 presents the average concentrations of metal fractions in the MSW compost sample with varying distributions for each metal fraction. From Fig. 8, certain general trends were observed.

The distribution of Cd, Cu, and Pb indicates the highest proportions of 33.84%, 70.87%, and 45.41%, while Zn has the second-highest proportion at 27.14% in the exchangeable fraction (F2) in the original sample. Metals in this fraction are held on the solid surface by weak electrostatic forces and could be released through ion-exchange processes. Factors such as changes in pH or ionic composition may affect remobilization from this fraction^53^. However, Zn showed a higher fraction in F6 (40.51%), suggesting that it is closely integrated with the crystalline lattice of MSW compost matrix, making it less easily extractable even with chemical treatment enhancements^73^. Table S8 indicates that a significant percentage of Cd (51.91%), Cu (87.30%), and Pb (66.86%) is weakly associated with F1, F2, and F3 fractions at levels exceeding 50% of the total. This finding supports previous research suggesting that these metals can be effectively removed using chelating agents^21,46^. The instability and susceptibility to environmental conditions of fractions F1, F2, and F3 make them prone to leaching into the environment. Several studies have highlighted that these fractions may accumulate in plants and pose risks to the food chain^21,32^.

Following GLDA-assisted leaching, the initial acid-extraction step in the form of carbonate is likely to be affected by environmental factors. The susceptibility of this fraction to pH changes may lead to increased release of metals in sludge when used as a soil fertilizer under acidic conditions such as acid rain^74^. The reducible fraction, also referred to as a heavy metal sink, primarily consists of elements bound within Fe − Mn oxides^21^. The primary presence of Cd, Cu, Pb, and Zn in the reducible portion may be attributed to the strong adsorption properties of non-crystalline Fe and Mn oxyhydroxides initially present in the exchangeable fraction. Over time, these can transform into less mobile forms specifically attached to surfaces. Metals found in the oxidizable fraction could result from a complexation or bioaccumulation process involving various types of organic matter such as organisms, detritus, or coatings on mineral particles. The preferential binding of divalent ions over monovalent ions exhibited by organic substances might explain why Cu, Pb and Zn are predominant in the oxidizable portion since metal ion binding strength order onto organic matter^45^ is likely Cu > Pb > Zn > Cd. Metals bound to organic matter are expected to persist in the MSW compost for extended periods compared to the reducible fraction, but they may be released by decomposition processes^19^. The post-leaching residual fraction F6 showed a substantial increase in Cd (46.74%), Cu (31.35%), Pb (51.66%), and Zn (65.93%), which is typically attributed to chemical elements within the mineral lattice and deemed resistant to environmental influences^21,47^. Therefore, the predominant association of Cd, Cu, Pb, and Zn with the residual fraction indicates their stability in MSW compost.

## Conclusions

Municipal solid waste poses a challenge as the economy and urban population continue to expand. Implementing a circular economy approach reduces waste by promoting the use of eco-friendly MSW compost. However, contamination of compost by heavy metals hinders the widespread adoption of MSW compost. This study suggests an environmentally friendly method for remediation of contaminated municipal solid waste compost using chemical-assisted leaching to remove heavy metals, along with geochemical fractionation pre- and post- treatment. GLDA (l-glutamic acid,*N*,*N*-diacetic acid) was proposed as a biodegradable complexing agent for heavy metal removal. The findings demonstrated that the levels of GLDA, pH, and retention time significantly affected the removal rates of Cd, Cu, Pb, and Zn. We utilized RSM model with the BBD design to optimize these factors. The optimization results suggested that in MSW compost, it was possible to achieve 90.32% removal for Cd, 81.96% for Cu, 91.62% for Pb, and 80.34% for Zn with a GLDA concentration of 150 mM at a pH of 2.9 over a retention time of 120 min. Also, an experiment comparing the traditional non-biodegradable chelator EDTA with the biodegradable GLDA was conducted under ideal conditions. The results showed that both achieved similar removal efficiency, highlighting the superior performance of GLDA due to its biodegradable nature.

Further, the study emphasizes the importance of understanding the geochemical distribution of metals in MSW compost. To examine the distribution of Cd, Cu, Pb, and Zn across different phases, we conducted sequential extraction of pre- and post-treated MSW compost. Cd, Cu, and Pb showed weak bonding in F1, F2, and F3 fractions—mainly found in the exchangeable fraction—which suggests potential remobilization by environmental factors. On the other hand, Zn demonstrated a distinct pattern with a notable presence in fraction F6; indicating strong integration with the crystalline lattice of the compost matrix that makes it less easily extractable even with chemical treatments. GLDA-assisted leaching changes the carbonate form, increasing Cu percentage. The reducible fraction composed of Fe–Mn oxides shows association with Cd, Cu, Pb, and Zn indicating transformation into less mobile forms over time. The oxidizable fraction linked to organic material demonstrates selectivity for divalent ions. After leaching, the residual fraction (F6) sees a significant increase in Cd, Cu, Pb, and Zn indicating stability within the compost's mineral lattice resistant to environmental factors.

Overall, the research discloses that GLDA shows promising potential for treating contaminated municipal solid waste compost and creating improved fertilizer for future agricultural use. It is important to evaluate the regeneration of GLDA in order to reduce remediation costs. Furthermore, deeper research is needed to understand changes in the elemental composition, surface chemistry, and colloidal structure of MSW compost after leaching.

### Supplementary Information

Supplementary Information.

## Data Availability

All data generated or analysed during this study are included in this published article [and its supplementary information files].
