# Supplementary material for: Bio-chelate assisted leaching for enhanced heavy metal remediation in municipal solid waste compost
Source: Sci Rep. 2024 Jun 20;14:14238. doi: 10.1038/s41598-024-65280-1 (PMC11190260; doi:10.1038/s41598-024-65280-1)
Supplement: Supplementary file 1 — Supplementary Information. [file 41598_2024_65280_MOESM1_ESM.docx]

**SUPPLEMENTARY MATERIAL**

**Supplementary material caption**

**Table S1.** Leaching effect of GLDA concentration on the removal efficiencies of Cd, Cu, Pb and Zn from MSW compost

**Table S2.** Leaching effect of pH on the removal efficiencies of Cd, Cu, Pb and Zn from MSW compost

**Table S3.** Leaching effect of retention time on the removal efficiencies of Cd, Cu, Pb and Zn from MSW compost

**Table S4.** Recovery methods for chelated metal-complex solution

**Table S5.** BBD matrix and experimental data showcasing metal removal efficiencies from MSW compost.

**Table S6.** Analysis of variance for the fitted quadratic model of Cd, Cu, Pb, and Zn removal efficiencies from MSW compost.

**Table S7.** Classification of MSW composts for their marketability and use in different areas

**Table S8.** Geochemical fractions of metals in pre- and post-leaching of MSW compost (Optimum condition: 150mM concentration of GLDA; 2.9 pH; and 120 mins retention time).

**Fig. S1.** Analytical conditions and chemical reagents for a modified Tessier's sequential extraction procedure.

**Fig. S2.** Heavy metal fractions in the modified sequential extraction process

**Fig. S3.** Pseudo-second-order kinetic models to the equilibrium data of Cd (a), Cu (b), Pb (c), and Zn (d).

**Fig. S4** Normal plot of studentized residuals versus normal % probability for Cd (a), Cu (b), Pb (c), and Zn (d) removal efficiencies, respectively.

**Fig. S5.** Correlation of observed and projected values and graphical representation of residuals plotted against projected values for metal removal from MSW compost.

**S1.** MSW compost physicochemical examination methods

**S2.** Kinetic Study

**Table S1.** Leaching effect of GLDA concentration on the removal efficiencies of Cd, Cu, Pb and Zn from MSW compost

| GLDA (mM) | Removal efficiency (%) | | | |
| --- | --- | --- | --- | --- |
|  | **Cd** | **Cu** | **Pb** | **Zn** |
| 1 | 4.59 ± 2.31 | 5.53 ± 2.33 | 1.15 ± 1.88 | 16.28 ± 1.05 |
| 5 | 11.47 ± 1.13 | 6.26 ± 2.68 | 2.22 ± 4.92 | 16.44 ± 1.98 |
| 10 | 20.64 ± 1.82 | 7.79 ± 1.65 | 4.08 ± 1.56 | 17.81 ± 2.67 |
| 15 | 29.82 ± 1.65 | 11.27 ± 2.16 | 7.73 ± 2.84 | 21.78 ± 2.18 |
| 20 | 38.99 ± 1.98 | 14.7 ± 2.99 | 12.6 ± 1.06 | 26.49 ± 1.48 |
| 40 | 48.17 ± 2.56 | 16.44 ± 1.65 | 17.76 ± 1.98 | 30.69 ± 1.36 |
| 60 | 55.05 ± 2.97 | 18.05 ± 1.84 | 19.69 ± 2.67 | 33.42 ± 2.49 |
| 80 | 59.63 ± 1.11 | 18.87 ± 1.32 | 18.97 ± 2.44 | 33.77 ± 2.24 |
| 100 | 61.1 ± 1.67 | 27.25 ± 1.06 | 26.92 ± 1.35 | 37.9 ± 2.44 |
| 120 | 70.28 ± 1.38 | 33.27 ± 2.49 | 33.51 ± 1.87 | 46.77 ± 1.84 |
| 140 | 77.45 ± 1.46 | 36.18 ± 2.85 | 38.59 ± 1.44 | 58.35 ± 1.34 |
| 160 | 80.04 ± 2.18 | 38.52 ± 2.04 | 43.03 ± 1.68 | 66.82 ± 1.87 |
| 180 | 81.33 ± 2.99 | 39.67 ± 1.95 | 46.61 ± 2.37 | 68.87 ± 1.11 |
| 200 | 81.71 ± 1.58 | 40.59 ± 1.32 | 48.69 ± 1.64 | 70.42 ± 2.46 |

Values represent mean ± standard deviation of three replicate

**Table S2.** Leaching effect of pH on the removal efficiencies of Cd, Cu, Pb and Zn from MSW compost

| pH | Removal efficiency (%) | | | |
| --- | --- | --- | --- | --- |
|  | **Cd** | **Cu** | **Pb** | **Zn** |
| 2 | 87.16 ± 2.49 | 53.23 ± 2.14 | 47.76 ± 2.56 | 58.6 ± 2.46 |
| 3 | 77.7 ± 3.44 | 38.66 ± 1.68 | 45.89 ± 2.77 | 49.05 ± 3.58 |
| 4 | 73.39 ± 1.68 | 29.21 ± 1.99 | 32.51 ± 2.09 | 39.55 ± 1.66 |
| 5 | 66.51 ± 2.57 | 22.08 ± 2.46 | 17.68 ± 1.36 | 25.94 ± 1.74 |
| 6 | 59.63 ± 2.98 | 15.08 ± 2.41 | 11.6 ± 2.42 | 20.65 ± 1.92 |
| 7 | 50.46 ± 2.46 | 13.56 ± 2.64 | 8.16 ± 2.67 | 19.8 ± 2.76 |
| 8 | 41.28 ± 3.59 | 12.9 ± 2.37 | 8.16 ± 2.43 | 17.97 ± 2.41 |
| 9 | 27.52 ± 3.74 | 6.21 ± 2.55 | 12.31 ± 2.85 | 16.12 ± 2.39 |
| 10 | 16.06 ± 2.94 | 5.31 ± 2.92 | 12.6 ± 2.03 | 13.17 ± 2.48 |

Values represent mean ± standard deviation of three replicates

**Table S3.** Leaching effect of retention time on the removal efficiencies of Cd, Cu, Pb and Zn from MSW compost

| Retention Time (mins) | Removal efficiency (%) | | | |
| --- | --- | --- | --- | --- |
|  | Cd | Cu | Pb | Zn |
| 5 | 27.52 ± 2.19 | 27.36 ± 1.22 | 21.42 ± 1.66 | 24.52 ± 2.19 |
| 10 | 32.11 ± 1.44 | 30.36 ± 3.59 | 22.12 ± 2.59 | 30.01 ± 2.38 |
| 20 | 34.17 ± 1.69 | 32.89 ± 2.66 | 26.67 ± 2.67 | 34.34 ± 2.66 |
| 30 | 40.55 ± 1.38 | 37.13 ± 2.87 | 38.17 ± 2.18 | 39.33 ± 2.75 |
| 40 | 48.91 ± 2.56 | 40.08 ± 2.19 | 40.22 ± 2.06 | 40.7 ± 2.19 |
| 50 | 51.1 ± 2.44 | 43.03 ± 2.06 | 44.54 ± 2.44 | 44.62 ± 1.95 |
| 60 | 67.69 ± 2.98 | 45.18 ± 2.48 | 51.71 ± 2.92 | 47.48 ± 2.67 |
| 90 | 80.5 ± 2.16 | 63.59 ± 2.34 | 67.19 ± 2.16 | 64.17 ± 2.03 |
| 120 | 83.03 ± 2.53 | 81.35 ± 1.26 | 86.07 ± 2.43 | 79.56 ± 2.64 |
| 240 | 84.17 ± 1.68 | 83.28 ± 1.98 | 89.16 ± 2.75 | 83.64 ± 2.94 |
| 360 | 85.09 ± 2.46 | 85.03 ± 2.67 | 90.36 ± 2.17 | 85.05 ± 2.15 |
| 480 | 86.7 ± 2.73 | 85.68 ± 2.15 | 90.88 ± 2.33 | 85.18 ± 2.63 |

Values represent mean ± standard deviation of three replicates

**Table S4.** BBD matrix and experimental data showcasing metal removal efficiencies from MSW compost.

| Assays | Independent variables | | | Metal removal efficiency (%) | | | |
| --- | --- | --- | --- | --- | --- | --- | --- |
|  | *k_1_* | *k_2_* | *k_3_* | Cd | Cu | Pb | Zn |
| 1 | -1(130) | -1(2.5) | 0(150) | 86.74 ± 1.49cd | 41.91 ± 1.22c | 43.98 ± 1.27cd | 52.96 ± 1.76de |
| 2 | 1(170) | -1(2.5) | 0(150) | 89.94 ± 1.44ab | 44.19 ± 2.02c | 43.02 ± 1.09cde | 61.38 ± 1.3a |
| 3 | -1(130) | 1(3.5) | 0(150) | 81.82 ± 1.47f | 33.82 ± 1.62e | 36.78 ± 1.61f | 47.01 ± 2.4de |
| 4 | 1(170) | 1(3.5) | 0(150) | 87.13 ± 2.41cd | 42.43 ± 1.72c | 45.45 ± 1.05c | 61.85 ± 1.86c |
| 5 | -1(130) | 0(3) | -1(120) | 83.94 ± 0.93ef | 73.03 ± 0.54ab | 78.34 ± 1.13ab | 75.7 ± 1.6de |
| 6 | 1(170) | 0(3) | -1(120) | 90.31 ± 2.02a | 74.57 ± 0.82a | 80.82 ± 1.46a | 76.05 ± 1.66ef |
| 7 | -1(130) | 0(3) | 1(180) | 85.09 ± 0.9de | 36.95 ± 1.17d | 41.97 ± 1.49de | 48.92 ± 1.59b |
| 8 | 1(170) | 0(3) | 1(180) | 87.9 ± 1.79bc | 37.14 ± 1.19d | 44.94 ± 1.09c | 62.16 ± 1.37c |
| 9 | 0(150) | -1(2.5) | -1(120) | 88.44 ± 0.5abc | 72 ± 1.62b | 77.24 ± 1.95b | 78.43 ± 0.86g |
| 10 | 0(150) | 1(3.5) | -1(120) | 86.77 ± 1.62cd | 74.94 ± 1.22a | 77.51 ± 2.09b | 76.4 ± 1.09ab |
| 11 | 0(150) | -1(2.5) | 1(180) | 88.67 ± 0.92abc | 42.71 ± 3.14c | 44.36 ± 1.6cd | 56.07 ± 1.71d |
| 12 | 0(150) | 1(3.5) | 1(180) | 85.39 ± 1.01de | 33.99 ± 1.68e | 38.46 ± 0.75f | 57.58 ± 1.97f |
| 13(C) | 0(150) | 0(3) | 0(150) | 86.86 ± 1.37cd | 35.56 ± 1.26de | 44.29 ± 1.61cd | 58.39 ± 1.51c |
| 14(C) | 0(150) | 0(3) | 0(150) | 87.79 ± 0.66bc | 37.72 ± 0.7d | 41.88 ± 2.47de | 56.14 ± 0.91ab |
| 15(C) | 0(150) | 0(3) | 0(150) | 87 ± 0.52cd | 36.19 ± 0.68de | 41.4 ± 0.85e | 55.48 ± 1.3g |

(C) represents central design points. Lowercase letters indicate statistical significance in results as per Fisher's protected LSD test at significance level p < 0.05.

**Table S5.** Analysis of variance for the fitted quadratic model of Cd, Cu, Pb, and Zn removal efficiencies from MSW compost.

| Source | DF* | Sum of squares | | | | F-value | | | | p-value | | | |
| --- | --- | --- | --- | --- | --- | --- | --- | --- | --- | --- | --- | --- | --- |
|  |  | Cd | Cu | Pb | Zn | Cd | Cu | Pb | Zn | Cd | Cu | Pb | Zn |
| Model | 9 | 66.96 | 3764.41 | 3892.93 | 1463.67 | 24.56 | 128.08 | 389.8 | 41.78 | 0.0013 | < 0.0001 | < 0.0001 | 0.0004 |
| *k_1_* | 1 | 39.25 | 19.88 | 21.68 | 169.92 | 129.57 | 6.09 | 19.54 | 43.65 | < 0.0001 | 0.0567 | 0.0069 | 0.0012 |
| *k_2_* | 1 | 20.1 | 30.54 | 13.49 | 4.5 | 66.34 | 9.35 | 12.16 | 1.16 | 0.0005 | 0.0282 | 0.0175 | 0.3314 |
| *k_3_* | 1 | 0.72 | 2581.93 | 2598.48 | 837.43 | 2.38 | 790.64 | 2341.66 | 215.13 | 0.1838 | < 0.0001 | < 0.0001 | < 0.0001 |
| *k_1_k_2_* | 1 | 1.1 | 10.05 | 23.23 | 10.34 | 3.64 | 3.08 | 20.94 | 2.66 | 0.1147 | 0.1398 | 0.006 | 0.1641 |
| *k_1_k_3_* | 1 | 3.17 | 0.4556 | 0.06 | 41.6 | 10.46 | 0.1395 | 0.0541 | 10.69 | 0.0231 | 0.7241 | 0.8253 | 0.0222 |
| *k_2_k_3_* | 1 | 0.64 | 34.05 | 9.58 | 3.15 | 2.11 | 10.43 | 8.63 | 0.8094 | 0.2058 | 0.0232 | 0.0323 | 0.4095 |
| *k_1_^2^* | 1 | 1.59 | 11.99 | 3.36 | 4.77 | 5.24 | 3.67 | 3.02 | 1.23 | 0.0707 | 0.1135 | 0.1425 | 0.3187 |
| *k_2_^2^* | 1 | 0.0785 | 19.39 | 5.11 | 0.2708 | 0.2592 | 5.94 | 4.61 | 0.0696 | 0.6323 | 0.0589 | 0.0846 | 0.8025 |
| *k_3_^2^* | 1 | 0.2385 | 1083.35 | 1201.74 | 382.52 | 0.7874 | 331.75 | 1082.96 | 98.27 | 0.4155 | < 0.0001 | < 0.0001 | 0.0002 |
| Residual | 5 | 1.51 | 16.33 | 5.55 | 19.46 |  |  |  |  |  |  |  |  |
| Lack of fit | 3 | 1.01 | 13.88 | 0.7515 | 14.82 | 1.34 | 3.79 | 0.1044 | 2.13 | 0.454 | 0.2158 | 0.9502 | 0.3357 |
| Pure error | 2 | 0.5029 | 2.44 | 4.8 | 4.64 |  |  |  |  |  |  |  |  |
| Core total | 14 | 68.48 | 3780.74 | 3898.48 | 1483.13 |  |  |  |  |  |  |  |  |
|  | Cd: R^2^ = 0.97; R^2^ _adj_ = 0.93; CV* (%) = 0.63; AP* = 18.09 | | | | | | | | | | | | |
|  | Cu: R^2^ = 0.98; R^2^ _adj_ = 0.93; CV (%) = 3.78; AP = 28.61 | | | | | | | | | | | | |
|  | Pb: R^2^ = 0.99; R^2^ _adj_ = 0.99; CV (%) = 2.02; AP = 51.29 | | | | | | | | | | | | |
|  | Zn: R^2^ = 0.98; R^2^ _adj_ = 0.96; CV (%) = 3.20; AP = 19.45 | | | | | | | | | | | | |

*DF: Degree of freedom; CV: Coefficient of variation; AP: Adequate precision

**Table S6.** Geochemical fractions of metals in pre- and post-leaching of MSW compost (Optimum condition: 150mM concentration of GLDA; 2.9 pH; and 120 mins retention time).

| Parameter | Cd | | Cu | | Pb | | Zn | |
| --- | --- | --- | --- | --- | --- | --- | --- | --- |
|  | Original | After leaching | Original | After leaching | Original | After leaching | Original | After leaching |
| Total Conc. | 21.8 | 2.11 | 1836.51 | 331.30 | 698.35 | 58.52 | 8130.73 | 1598.50 |
| F1 | 0.77 ± 0.03 | 0.01 ± 0.01 | 39.77 ± 2.09 | 1.13 ± 0.06 | 33.37 ± 2.36 | 0.05 ± 0.04 | 44.75 ± 8.91 | 1.01 ± 0.1 |
| F2 | 7.37 ± 0.06 | 0.36 ± 0.01 | 1301.48 ± 9.39 | 92.3 ± 0.62 | 317.1 ± 8.44 | 16.87 ± 0.25 | 2206.69 ± 52.3 | 112.59 ± 0.66 |
| F3 | 3.17 ± 0.12 | 0.08 ± 0.01 | 262 ± 5.4 | 62.39 ± 0.3 | 116.45 ± 4.74 | 11.9 ± 0.89 | 1221.46 ± 45.16 | 94.78 ± 1.93 |
| F4 | 5.2 ± 0.06 | 0.66 ± 0.01 | 36.78 ± 0.45 | 21.09 ± 0.16 | 39.2 ± 3.12 | 17.55 ± 1.17 | 379.1 ± 14.06 | 77.66 ± 2.21 |
| F5 | 3.28 ± 0.15 | 0.01 ± 0 | 52.7 ± 1.97 | 31.57 ± 0.92 | 49.93 ± 4.28 | 17.15 ± 1.12 | 896.56 ± 17.41 | 156.51 ± 4.46 |
| F6 | 1.75 ± 0.06 | 0.92 ± 0.01 | 133.52 ± 4.88 | 121.17 ± 0.92 | 137.13 ± 2.84 | 68.76 ± 0.45 | 3294.08 ± 185.74 | 1129.93 ± 54.91 |
| ^a^SEE (%) | 98.87 | 98.26 | 99.44 | 99.51 | 99.26 | 99.65 | 98.92 | 98.37 |

^a^SEE is sequential extraction efficiency defined as the sum of all fractions divided by the total metal content.

**Table S7.** Classification of MSW composts for their marketability and use in different areas**^12^**

| Class | | Fertilizing index | Clean  index | Quality control compliance | Remark |
| --- | --- | --- | --- | --- | --- |
| Marketable | A | > 3.5 | > 4.0 | Complying for all heavy metal  parameters | *Best quality.*  High manurial value potential and low heavy metal content and can be used for high value crops, like in organic farming. |
|  | B | 3.1 – 3.5 | > 4.0 | Complying for all heavy metal  parameters | *Very good quality.*  Medium fertilizing potential and low heavy metal content. |
|  | C | > 3.5 | 3.1 – 4.0 | Complying for all heavy metal  parameters | *Good quality.*  High fertilizing potential and medium heavy metal content. |
|  | D | 3.1 – 3.5 | 3.1 – 4.0 | Complying for all heavy metal  parameters | *Medium quality.*  Medium fertilizing potential and medium heavy metal content. |
| Restricted use | RU1 | < 3.1 | - | Complying for all heavy metal  parameters | Should not be allowed to market due to low fertilizing potential. However, these can be used as soil conditioner. |
|  | RU2 | >3.5 | >4.0 | Not complying for all heavy metal  parameters | Should not be allowed to market. Restricted use. Can be used for growing non-food crops. Requires periodic monitoring of soil quality if used repeatedly. |
|  | RU3 | >3.5 | <4.0 | Not complying for all heavy metal  parameters | Restricted use. Should not be allowed to market. Can be used only for developing lawns/gardens (with single application), rehabilitation of degraded land. |

**Table S8.** Recovery methods for chelated metal-complex solution

| S.No. | Treatment | Observation | Reference |
| --- | --- | --- | --- |
| 1 | [Electrodialysis](https://www.sciencedirect.com/topics/chemical-engineering/electrodialysis) (ED) combined with ion-exchange resins | The method included ongoing electropermutation in which metal cations bound to the resin were replaced by protons from the anodic compartment and moved to a receiving compartment at the cathodic side for concentration. Even with loaded resins with metallic cations before introducing them into the cell's feed compartment, high levels of removal were attained: 98% for Cd, 92.5% for Cu, 99% for Pb, and 95% for Zn. | 1, 2 |
| 2 | Solid phase extraction (SPE) system with an ion-selective immobilized macrocyclic material | This method is known as a molecular recognition technology (MRT)). Author studies the separation process for the recovery of metals from washing effluent containing excess aminopolycarboxylate chelant in solution. The recoveries observed were as follows: As: 99-101%; Cd: 84-102%; Cr: 101-102%; Pb: 98-100%; Se: 88-100%. | 3 |
| 3 | Ozone/percarbonate (O_3_/SPC) process | The findings indicate that the O_3_/SPC method achieves 100% Cu recovery and shows exceptional metal retrieval from other metal-organic compound wastewaters. In this technique, the carbonate radical anion (CO_3_•–) is produced through the O_3_/SPC process and initiates a targeted attack on amino groups of metal complexes to facilitate decarboxylation and deamination reactions. The recovered metal can be converted into stable precipitates with the help of an internal precipitant (CO_3_^2-^), resulting in non-toxic byproducts in the O_3_/SPC process. | 4 |
| 4 | Neutral pH electro-Fenton (EF) system | This method decomplexes 99.9% of copper-organic bonds within 8 hours. At the same time, the resulting copper ions are directly deposited onto the carbon cloth cathode as pure Cu nanoparticles with a purity exceeding 95.0%. This neutral approach enables the simultaneous removal of Cu, Ni, and Cr complexes through an efficient electrochemical process involving an iron anode, carbon cloth cathode, and sodium tetrapolyphosphate electrolyte (Na_6_TPP). | 5 |
| 5 | Discharge Plasma Oxidation method coupled Alkaline Precipitation for metal removal | The decomplexation efficiency of Cu complex reached up to nearly 100% after 60 mins oxidation by discharge plasma. The followed precipitation process removed 78.1% of Cu2+, and Cu-associated precipitates included CuCO_3_, Cu_2_CO_3_(OH)_2_, CuO, and Cu(OH)_2_. | 6 |
| 6 | Sulfide precipitation using Na_2_S | Metal sulfide precipitates are produced in this procedure, leading to the retrieval of the chelating agent. This method demonstrates rapid reaction rates and achieved a Cd removal efficiency of 99.99 ± 0.001%. The dechelation process was finished in under 20 minutes, and the reclaimed chelating agent maintains its capacity to extract heavy metals after acidification. | 7 |
| 7 | TiO_2_photocatalysis methods | TiO_2_ is commonly employed in the treatment of heavy metal complex-containing wastewater, as it has the capacity to fully mineralize organic ligands under mild conditions (such as room temperature and atmospheric pressure). This feature makes it highly promising for extensive industrial use. | 8, 9 |
| 8 | Fenton-like oxidation process | The Fenton reaction involves the interaction of soluble iron cations with H_2_O_2_ to produce radical –•OH, which can be utilized for breaking down metal organic matter complexes and releasing free metal ions. This method offers improved removal efficiency and is a conventional approach for treating chelated heavy metal wastewater. Its advantages include high activity, fast reaction rates, mild reaction conditions, and effective compliance with discharge effluent water quality standards. However, its functionality is limited to a narrow pH range. | 9, 10, 11 |


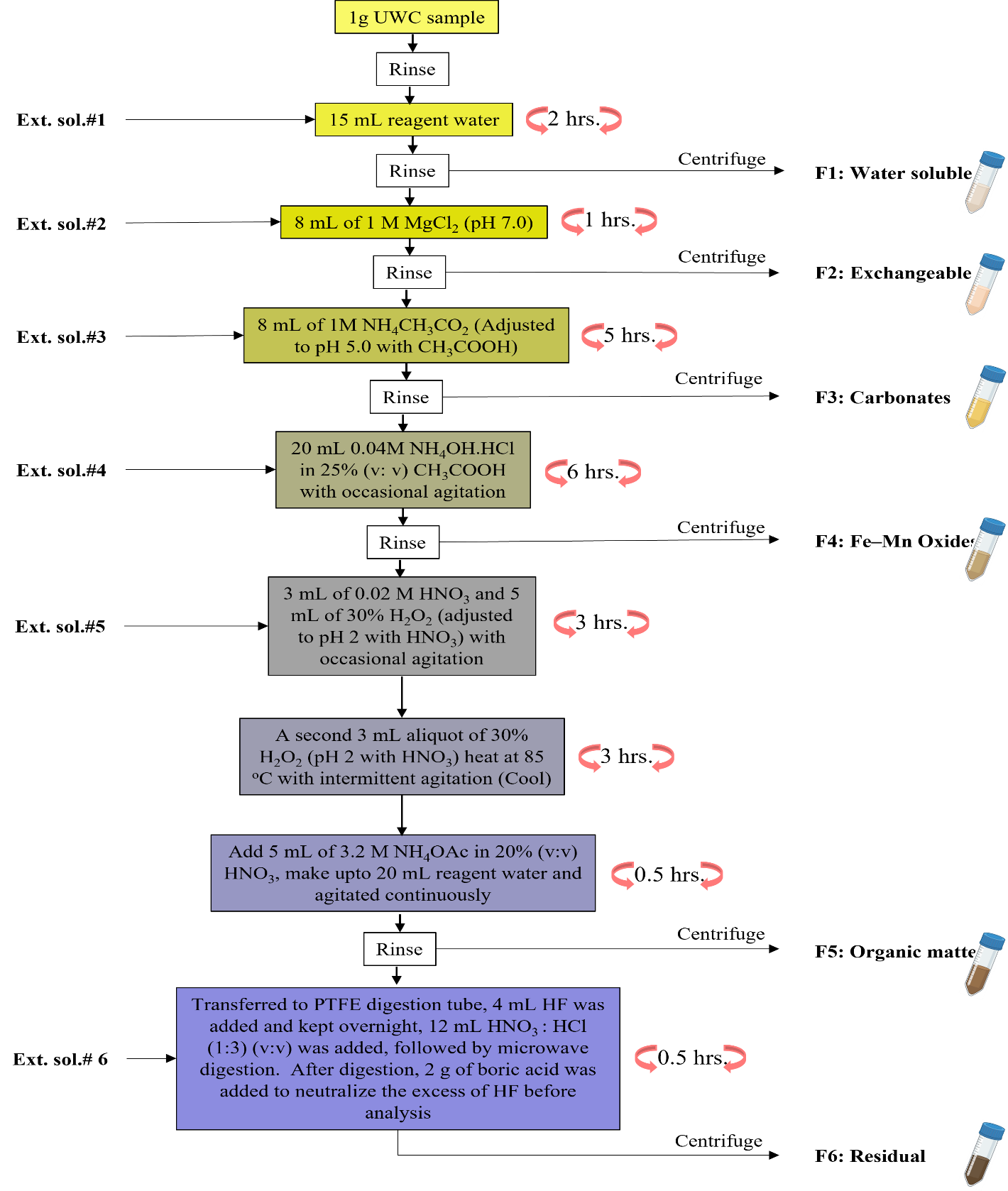


(Ext. sol.: Extraction solution); (Rinse with 8mL water, shake at 30rpm, centrifuge at 3000rpm for 30 mins.)

**Fig. S1.** Analytical conditions and chemical reagents for a modified Tessier's sequential extraction procedure.


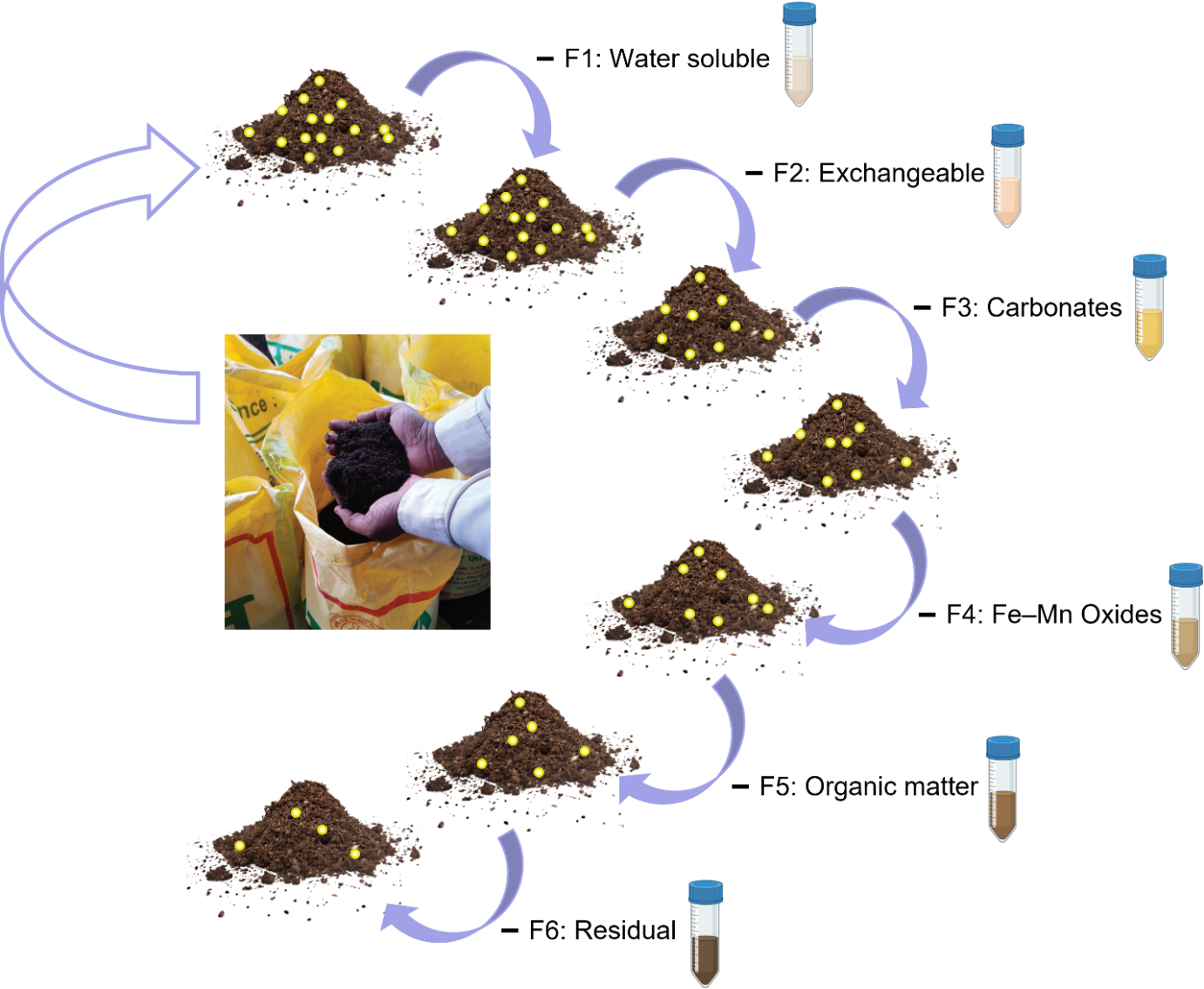


**Fig. S2.** Heavy metal fractions in the modified sequential extraction process

| 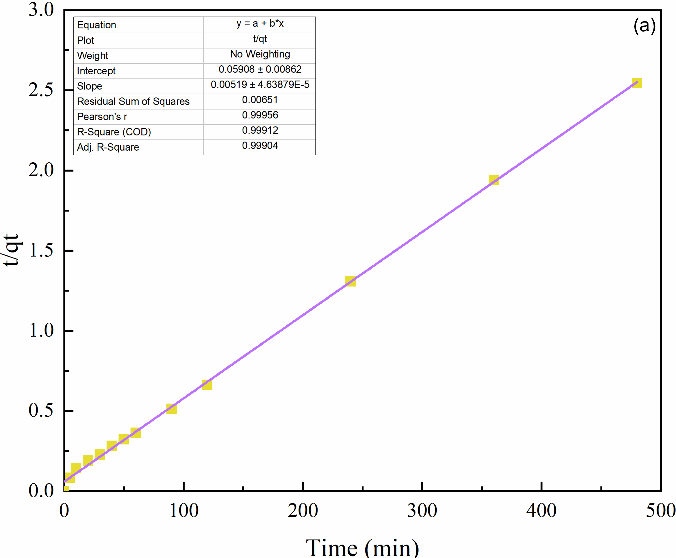 | 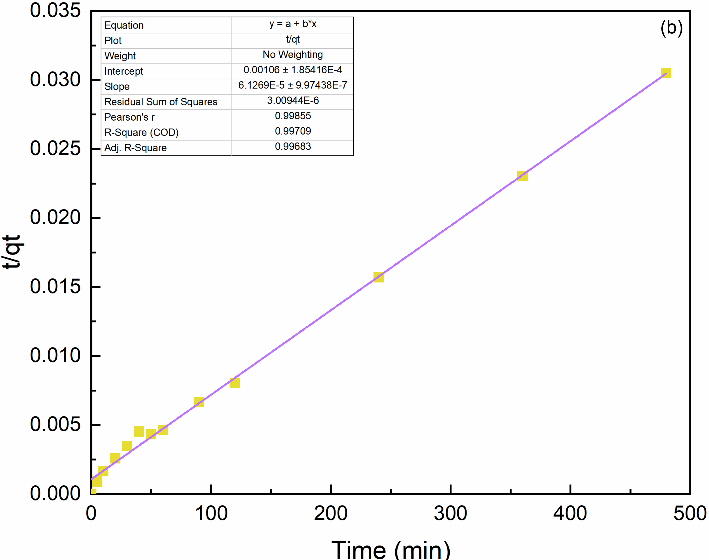 |
| --- | --- |
| 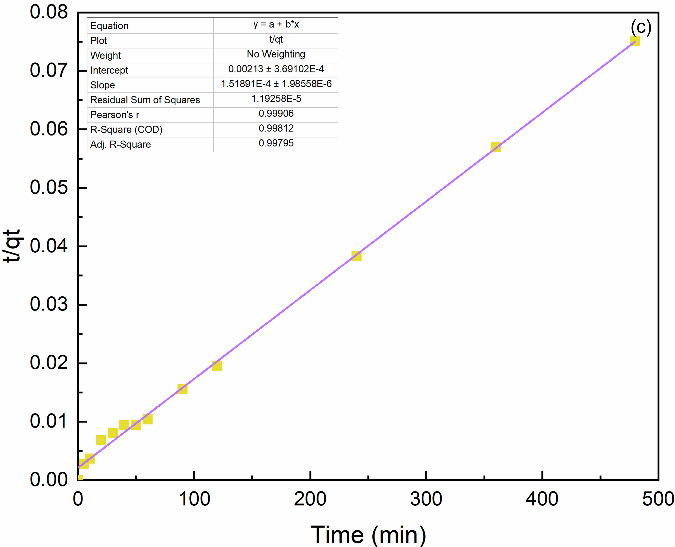 | 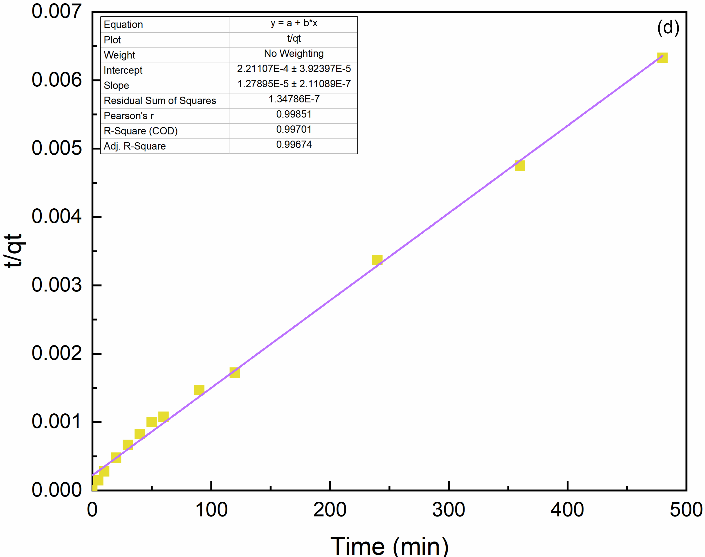 |

**Fig. S3.** Pseudo-second-order kinetic models to the equilibrium data of Cd (a), Cu (b), Pb (c), and Zn (d).


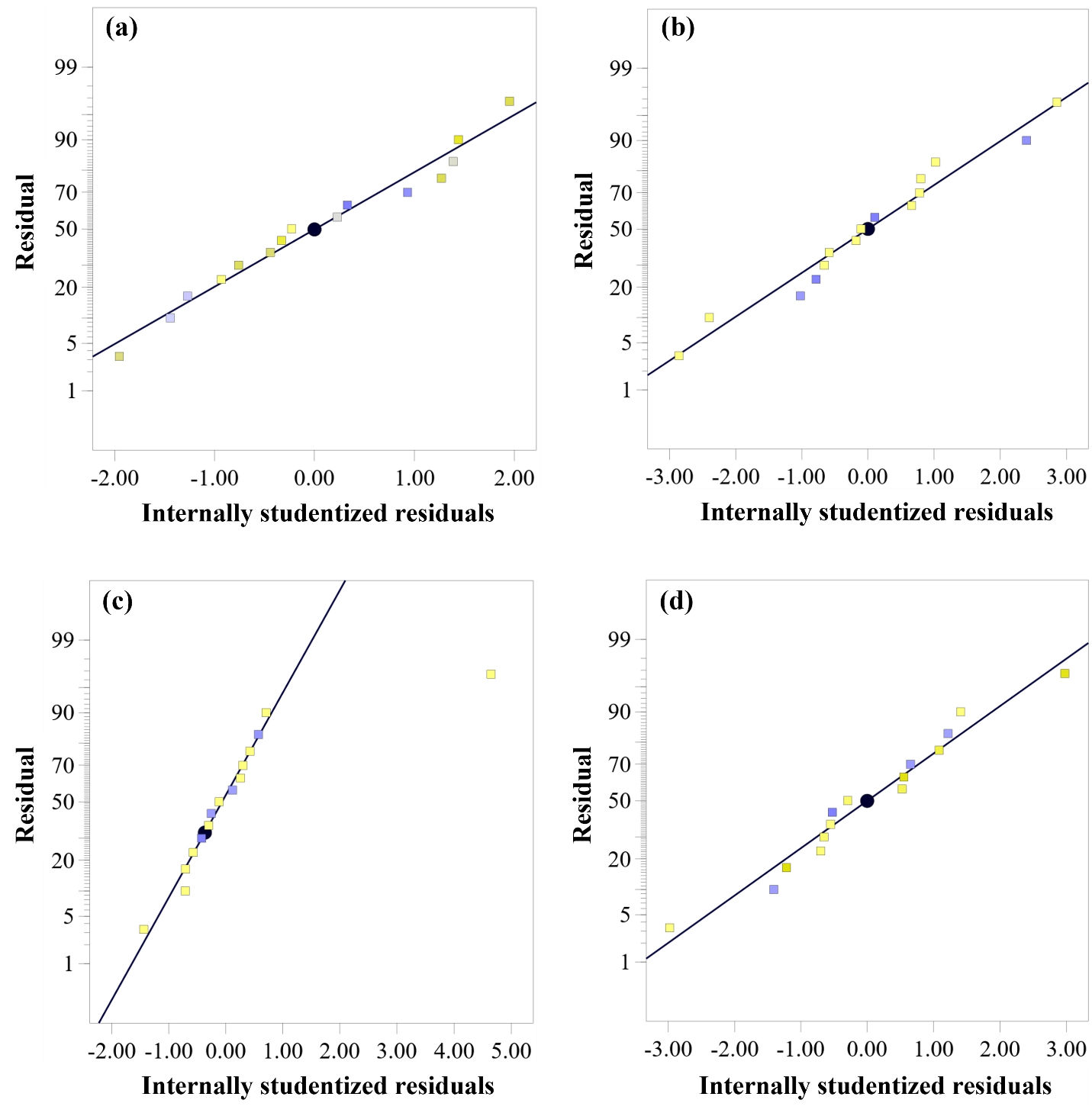


**Fig. S4.** Normal plot of studentized residuals versus normal % probability for Cd (a), Cu (b), Pb (c), and Zn (d) removal efficiencies, respectively.

| 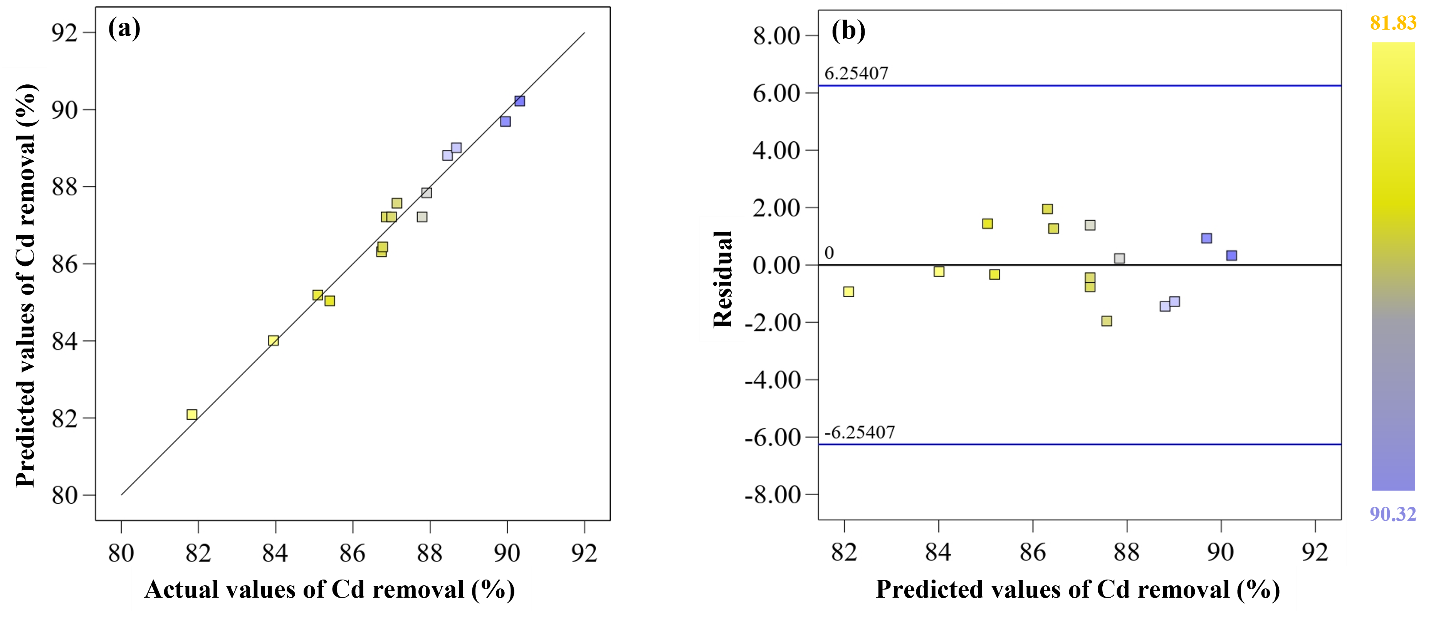 |
| --- |
| 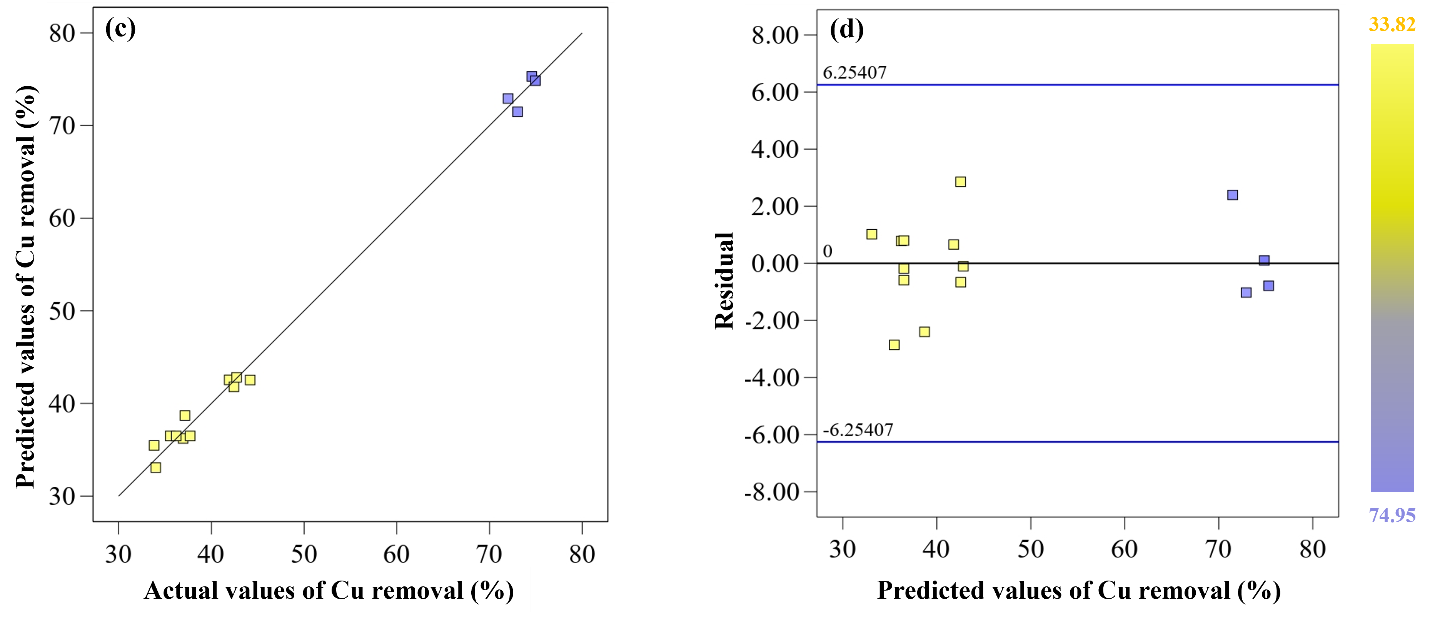 |
| 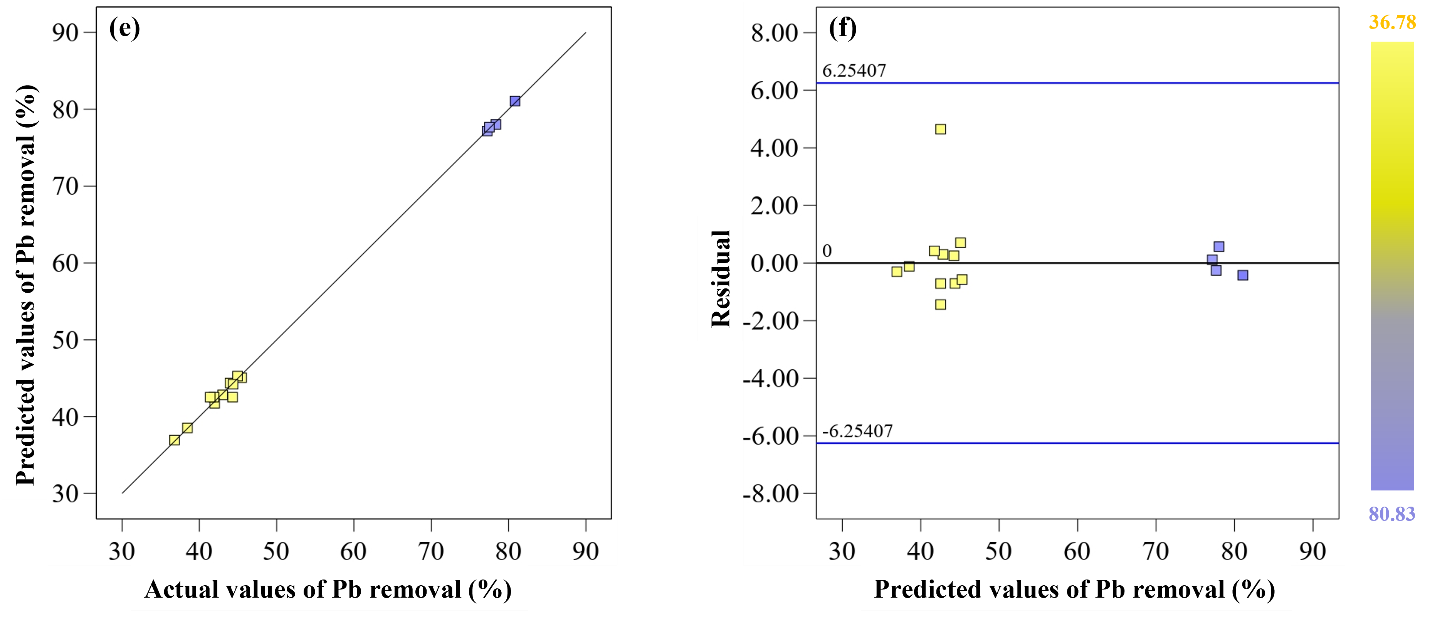 |
| 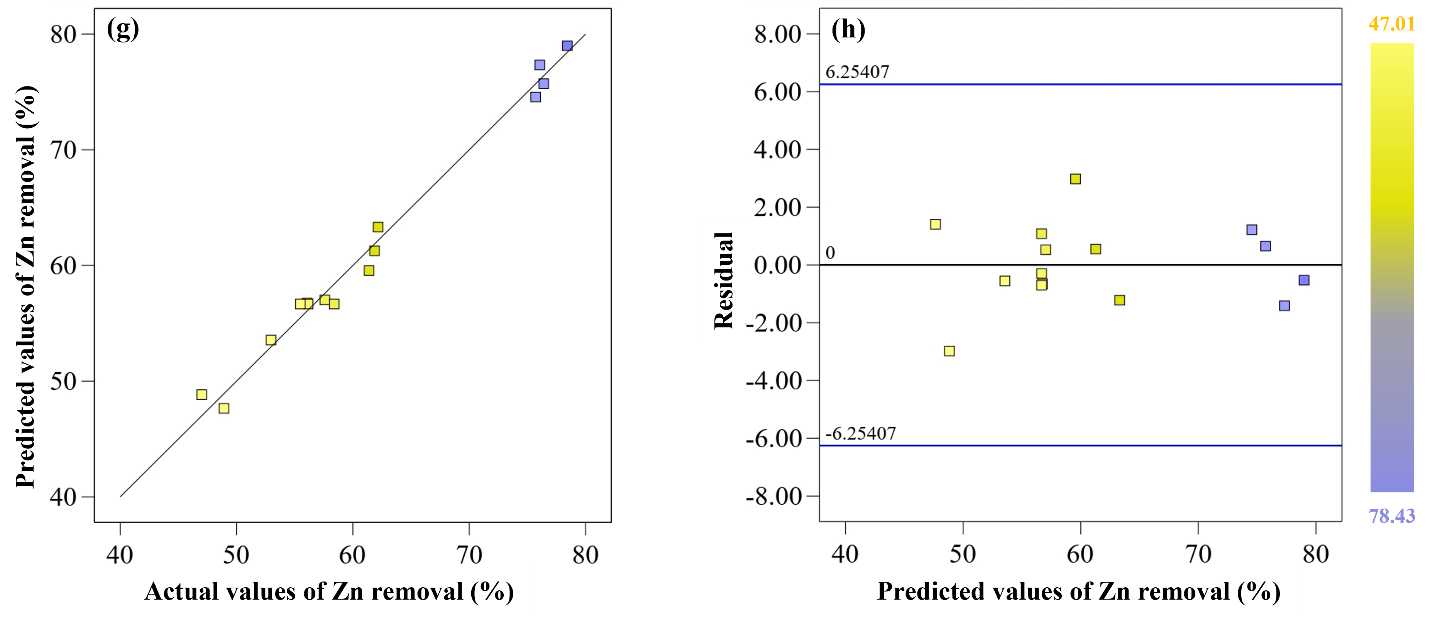 |
| **Fig. S5.** Correlation of observed and projected values and graphical representation of residuals plotted against projected values for metal removal from MSW compost. |

**S1. MSW compost physicochemical examination methods**

For the analysis, the sample was initially air-dried in the open air, sieved (2mm) and then finely ground to pass through a 0.25 mm mesh sieve after which it was stored in airtight containers.

***Physical analysis***

The gravimetric moisture content of the fresh samples was ascertained by observing weight loss over a 24-hour period at 70 °C. The samples were pulverized, filtered through a 4-mm sieve, and dried at 70 °C before the chemical characteristics were examined. Volatile solids (VS) were determined by measuring the mass loss during dry combustion for 5 hours at 550 ^o^C in a muffle furnace. Total organic carbon (TOC) percentage = (% VS) /1.724 is the formula used to determine TOC in accordance with IS 16556:2016, also pH and electrical conductivity of a slurry made from the dried sample with a water-to-sample ratio of 1:5 were measured.

***Chemical analysis***

To determine the amounts of total nitrogen (TN), total phosphorus (TP), total potassium (TK), and heavy metals in UWC, the following procedures have been utilized, as recommended by IS16556:2016:

1. Decomposing materials in a Kjeldahl flask with concentrated H_2_SO_4_ and a catalyst mixture allowed for the determination of TN. Following this, the TN content of the digest was determined through steam distillation and titration.

2. Following thorough digestion of the compost in a di-acid mixture (HClO_4_: HNO_3_ at a 9:4 ratio), TP was determined using the vanadomolybdate yellow colour method and spectrophotometer analysis.

3. The samples were burnt at 550 °C, dissolved in HCl, and the total potassium (TK) analysis was performed using a flame photometer equipped with a K-filter to determine the extract's K content.

4. The samples were pulverized and sieved through a 0.2-mm sieve to estimate the quantities of heavy metals (Cd, Cu, Cr, Pb, Ni, and Zn). Then, in a Teflon beaker, they were digested using a 10:1 diacid mixture (for Pb alone) and a triacid mixture (HNO_3_:H_2_SO_4_:HClO_4_). The extracts' composition for Cd, Cu, Cr, Pb, Ni, and Zn were examined using the AVIO200 ICP-OES.

**S2** **Kinetic study**

To ascertain the response rate and equilibrium time of the desorption process, an investigation was conducted on the kinetics of desorption. The kinetic model of extraction efficiency for heavy metals is of significant importance for better understanding the leaching mechanism. Two kinetic models have been applied to the kinetic data in order to examine the kinetic mechanism involved in the leaching process: the pseudo-first-order (Eq. S1) and pseudo-second-order (Eq. S2). The fitting of these two kinetic models to the equilibrium data of Cd, Cu, Pb, and Zn is presented in Fig. S2(a-d).

$\ln\left( q_{e}-q_{t} \right)=\ln q_{e}-k_{1}t$ Eq. (S1)

$\frac{t}{q_{e}}=\frac{1}{k_{2}q_{e}^{2}}+\frac{1}{q_{e}}$ Eq. (S2)

Where, $q_{e}$ and $q_{t}$ represent the concentrations of heavy metal ions (mg kg^-1^) at equilibrium and at time t, respectively. The equilibrium rate constant of the pseudo-first-order and pseudo-second order model is denoted as k_1_, and k_2_ represents the rate constant at equilibrium.

References:

1. Smara, A., Delimi, R., Poinsignon, C., Sandeaux, J. Electroextraction of heavy metals from diluted solutions by a process combining ion-exchange resins and membranes. *Separation and Purification Technology*, **44**(3), 271-277 (2005).
2. Juve, J. M. A., Christensen, F. M. S., Wang, Y., Wei, Z. Electrodialysis for metal removal and recovery: A review. *Chemical Engineering Journal*, **435**, 134857 (2022).
3. Hasegawa, H. et al. Recovery of toxic metal ions from washing effluent containing excess aminopolycarboxylate chelant in solution. *Water research*, **45**(16), 4844-4854 (2011).
4. Chen, Y. et al. Targeted decomplexation of metal complexes for efficient metal recovery by ozone/percarbonate. *Environmental Science & Technology*, **57**(12), 5034-5045 (2023).
5. Li, M. et al. An electrochemical strategy for simultaneous heavy metal complexes wastewater treatment and resource recovery. *Environmental Science & Technology*, **56**(15), 10945-10953 (2022).
6. Wang, T. et al. Novel Cu (II)–EDTA decomplexation by discharge plasma oxidation and coupled Cu removal by alkaline precipitation: underneath mechanisms. *Environmental science & technology*, **52**(14), 7884-7891 (2018).
7. Wang, Q., Chen, J., Zheng, A., Shi, L. Dechelation of Cd-EDTA complex and recovery of EDTA from simulated soil-washing solution with sodium sulfide. *Chemosphere*, **220**, 1200-1207 (2019).
8. Cho, I. H., Lee, N. H., Yang, J. K., Lee, S. M. Treatment of wastewater containing Cu (II)-EDTA using immobilized TiO2/solar light. *Journal of Environmental Science and Health Part A*, **42**(2), 165-170 (2007).
9. Zhu, Y., Fan, W., Zhou, T., Li, X. Removal of chelated heavy metals from aqueous solution: A review of current methods and mechanisms. *Science of the Total Environment*, **678**, 253-266 (2019).
10. Fu, F., Xie, L., Tang, B., Wang, Q., Jiang, S. Application of a novel strategy—Advanced Fenton-chemical precipitation to the treatment of strong stability chelated heavy metal containing wastewater. *Chemical Engineering Journal*, **189**, 283-287 (2012).
11. Fontmorin, J. M., Sillanpää, M. Dewatering and removal of metals from urban anaerobically digested sludge by Fenton’s oxidation. *Environmental technology*, **38**(4), 495-505 (2017).
12. Saha, J. K., Panwar, N., Singh, M. V. An assessment of municipal solid waste compost quality produced in different cities of India in the perspective of developing quality control indices. Waste Manag. 30, 192–201. https://doi.org/10.1016/j.wasman.2009.09.041 (2010).
